# Health Canada advisory impacts on the prevalence of oral codeine use in the Pediatric Canadian population: comparative study across provinces

**DOI:** 10.1038/s41598-024-55758-3

**Published:** 2024-03-04

**Authors:** O. Sheehy, S. Eltonsy, S. Hawken, M. Walker, P. Kaul, B. Winquist, O. Barrett, A. Savu, R. Dragan, M. Pugliese, S. Bernatsky, J. Gorgui, A. Bérard

**Affiliations:** 1 CHU Sainte-Justine, Research Center, 3175, Côte-Sainte-Catherine, Montreal, QC H3T 1C5 Canada; 2 Rady Faculty, College of Pharmacy, University of Manitoba, Winnipeg, MB Canada; 3 Clinical Epidemiology Program, Ottawa Hospital Research Institute, Ottawa, ON Canada; 4 Scholl of Epidemiology and Public Health, University of Ottawa, Ottawa, ON Canada; 5 ICES, Ottawa, ON Canada; 6 Better Outcomes Registry and Network (BORN) Ontario, Children’s Hospital of Eastern Ontario, Ottawa, ON Canada; 7 Departement of Obstetrics and Gynecology, University of Ottawa, Ottawa, ON Canada; 8 Department of Obstetrics, Gynecology and Newborn Care, The Ottawa Hospital, Ottawa, ON Canada; 9 International and Global Health Office, University of Ottawa, Ottawa, ON Canada; 10 Department of Medicine Faculty of Medicine and Dentistry, University of Alberta, Edmonton, AB Canada; 11 Department of Community Health and Epidemiology, College of Medicine, University of Saskatchewan, Saskatoon, Canada; 12 Data and Analytics, Alberta Health Services, Calgary, AB Canada; 13 Manitoba Centre for Health Policy, Winnipeg, MB Canada; 14 Ottawa Hospital Research Institute, ICES uOttawa, Ottawa, ON Canada; 15 Faculty of Medicine, McGill University, Montreal, QC Canada; 16 Faculty of Pharmacy, University of Montreal, Montreal, QC Canada; 17 Faculty of Medicine, Université Claude Bernard Lyon 1, Lyon, France

**Keywords:** Diseases, Health care, Medical research, Risk factors

## Abstract

Health Canada (HC) has, since 2013, issued safety alerts restricting the use of codeine-containing drugs among breastfeeding women and children/adolescents under 18 years of age. These products are linked to breathing problems among ultra-rapid CYP2D6 metabolizers and early use of opioid can lead to future opioid misuse. Using a multi-province population-based cohort study, we estimate the impact of federal safety alerts on annual rates of codeine use in the Canadian pediatric population. We analyzed data from 8,156,948 children/adolescents in five Canadian provinces between 1996 and 2021, using a common protocol. Children/adolescents were categorized as: ≤ 12 years (children) or > 12 years (adolescents). We defined codeine exposure by ≥ 1 prescription filled for codeine alone or combined with other medications. For both age categories, we obtained province-specific codeine prescription filling rates per calendar year by dividing the number of children/adolescents with ≥ 1 codeine prescription filled by the number of person-time. Annual rates of codeine use per 1000 persons vary by province from 3.0 (Quebec) to 10.1 (Manitoba) in children, and from 5.5 to 51.3 in adolescents. After the 2013 HC advisory, exposure decreased in all provinces (adjusted level change from − 0.6 to − 18.4%) in children and from − 2.1 to − 17.9% in adolescents after the 2016 advisory. Annual rates declined over time in all provinces, following HC safety alerts specific to each of the two age categories.

## Introduction

Codeine is an opioid analgesic that has been used for many years to relieve pain^1–9^ and reduce coughing, a symptom common in children^10–16^. Though codeine is considered a cough suppressant, its efficacy compared to placebo is unclear^15–23^. Since 2008, Health Canada (HC) issued safety alerts regarding adverse reactions associated with prescribed codeine medications^22^. In June 2013, HC reviewed the safety of medications containing codeine for pain or cough treatment and recommended against their use in children ≤ 12 years of age^23^. New safety measures for codeine prescriptions restricting their use in children/adolescents (< 18 years old) were published in July 2016^24^.

The history of safety alerts regarding the use of medication containing codeine in the pediatric population worldwide is summarized in Table 1.

**Table 1:** Worldwide Safety Alerts for codeine use.

Date	Country	Safety alert
2012–08-16	US FDA	Codeine use in certain children after tonsillectomy and/or adenoidectomy may lead to rare, but life-threatening adverse events or death (Letter to Healthcare Professionals)
2012–08-16	US FDA	FDA Drug Safety Communication: Safety review update of codeine use in children; new Boxed Warning and Contraindication on use after tonsillectomy and/or adenoidectomy
2013–02-21	US FDA	FDA Drug Safety Communication: Safety review update of codeine use in children; new Boxed Warning and Contraindication on use after tonsillectomy and/or adenoidectomy
2013–06-07		Codeine—Recommends only be used in patients aged 12 and over (Letters to Healthcare Professionals)
2013–06-07	Canada	Health Canada’s review recommends codeine only be used in patients aged 12 and over
2013–06-15	European Union	Pharmacovigilance Risk Assessment Committee (PRAC) recommends restricting the use of codeine when used for pain relief in children
2013–06-29	United Kingdom	Updates on the use of codeine. The MHRA confirmed that codeine-containing medicines should only be used in children over 12 years old to treat acute (short lived) moderate pain, and only if it cannot be relieved by other painkillers such as paracetamol or ibuprofen, following completion of a European review
2014–04-12	European Union	Start of review of codeine-containing medicines when used for cough and cold in children
2014–09-05	Singapore	Health Sciences Authority (HAS)overseas recommendations on the use of codeine-containing products for pain relief in paediatric patients
2015–03-14	European Union	PRAC recommends restrictions on the use of codeine for cough and cold in children
2015–04-25	European Union	Codeine not to be used in children below 12 years for cough and cold
2015–04-30	United Kingdom	Codeine for cough and cold: restricted use in children
2015–07-02	US	Codeine cough-and-cold medicines in children: Drug Safety Communication—FDA evaluating potential risk of serious side effects
2016–07-05	Singapore	Recommendations on the use of codeine-containing products for treatment of pain and relief of cough and cold in children and adolescents
2016–07-29	Canada	New safety measures for prescription codeine and hydrocodone to further restrict use in children and adolescents
2016–12-20	Australia	Update on the proposal for the rescheduling of codeine products—Codeine containing medicines to move to prescription only
2016–12-21	Singapore	Restrictions on the use of codeine-containing products in children and adolescents
2017–01-05	China	CFDA announcement on revision of package insert of medicines containing Codeine (Issue no. 199, 2016)
2017–04-21	US FDA	Prescription codeine pain and cough medicines and tramadol pain medicines: FDA restricts use in children; recommends against use in breastfeeding women (Letter to Healthcare Professionals)
2017–04-21	US FDA	FDA restricts use of prescription codeine pain and cough medicines and tramadol pain medicines in children; recommends against use in breastfeeding women
2017–11-29	Australia	Safety review: Codeine use in children and ultra-rapid metabolisers: Update—recommendations implemented
2018–01-12	US FDA	FDA requires labeling changes for prescription opioid cough and cold medicines to limit their use to adults 18 years and older (Letter to Healthcare Professionals)
2018–01-12	US FDA	FDA requires labeling changes for prescription opioid cough and cold medicines to limit their use to adults 18 years and older
2018–09-07	China	Announcement of the National Medical Products Administration on Revising the Instructions for Cold Medicines Containing Codeine (No. 63, 2018)
2019–02-19	Canada	Summary Safety Review—Opioid-containing cough and cold products—Assessing the potential risk of opioid use disorder and related harms in children and adolescents
2020–08-01	Canada	Non-prescription pain relief products containing codeine are not recommended for use in people under 18 years of age
2020–08-25	Canada	Prescription cough and cold products containing opioids and the risk of opioid use disorder in children and adolescents

Since the publication of safety reports on oral medications containing codeine use in children/adolescents, its use has become questionable even for pain management. A significant decrease in their use has been observed in the children/adolescents in the United States, with a 90.1% decrease in the use of cough medications containing codeine between 2014 and 2019^4,11,13,25^. In 2018, Canada ranked second in the world for per capita opioid use despite a 10% decrease between 2016 and 2017^26^. Data on trends and prevalence of codeine use in Canadian children/adolescents are lacking and differences in its use between provinces has not been evaluated. This is relevant given that the healthcare system is managed by provinces, which can result in differences in medication availability and prescribing/utilisation patterns across Canada. Additionally, the impact of Canadian safety warnings on codeine use among children/adolescents remains unknown. Further information on trends of use by age category, daily and cumulative dose, prescriber speciality, and indication is also needed.

The primary objective of this study was to quantify the impact of HC safety alerts on the annual rates of children exposed to codeine (alone or in combination with other medications), among Canadian children/adolescents within five provinces over the last decades using an interrupted time series (ITS) analysis. Therefore, our hypothesis is that HC warnings on codeine have reduced the number of codeine prescriptions in the Canadian pediatric population. We also investigated indications for filled codeine prescriptions, specialities of prescribers, and daily and cumulative dosage of codeine filled prescription.

## Results

### Overall rates of codeine exposure

Of the 8,156,948 children/adolescents included in the five provincial cohorts, 51.3% were male, and the weighted average follow-up was of 7.2 years with a standard deviation (SD) of 4.1. In all provinces, older children/adolescents (> 12) were more exposed to codeine than younger ones (≤ 12) (Table 2). Overall rates of children exposed to codeine (per 1000 children/-years) for both age categories were significantly higher in Manitoba (10.1, 95% CI 10.0–10.2 for children ≤ 12 years of age; and 51.3, 95%CI 51.0–60.5 for adolescents > 12 years of age, Table 2), and were the lowest in Quebec (3.0, 95% CI 2.9–3.1 for children ≤ 12 years of age; and 7.5, 95%CI 6.8–8.2 for adolescents > 12 years of age, Table 2).

**Table 2:** Characteristic and codeine exposure among children/ adolescents by age categories and provinces.

	Alberta	Saskatchewan	Manitoba	Ontario	Quebec^a^
	2004–2021	1998–2020	1996–2019	2012–2020	1999–2015
Number of live births	20,60,079	7,24,428	8,20,867	43,13,792	2,37,782
Follow-up time – mean ± sd	8.0 ± 5.0	12.9 ± 6.0	8.6 ± 5.7	5.7 ± 3.0	5.6 ± 4.2
Child/adolescent male gender – n (%)	1,056,820 (51.3)	370,762 (51.2)	421,105 (51.3)	2,212,975 (51.3)	12,222 (51.4)
Overall rates per 1000 children-year and age category (95% CI)					
Number of children with at least one filled codeine prescription					
Children ≤ 12 years of age	9.69 (9.63–9.75)	3.25 (3.20–3.31)	10.09 (10.00–10.18)	4.00 (3.97–4.03)	2.98 (2.89–3.08)
Adolescents > 12 years of age	40.27 (40.09–40.44)	19.31 (19.12–19.50)	51.31 (51.03–51.60)	29.71 (29.60–29.83)	7.49 (6.83–8.15)
Number of filled codeine prescriptions					
Children ≤ 12 years of age	11.18 (11.11–11.24)	3.35 (3.29–3.41)	11.52 (11.42–11.62)	4.77 (4.73–4.80)	3.41 (3.31–3.51)
Adolescents > 12 years of age	47.10 (46.92–47.29)	19.35 19.16–19.54)	62.01 (61.70–62.33)	32.31 (32.19–32.43)	8.46 (7.77–9.16)
Number of codeine treatment courses					
Children ≤ 12 years of age	10.90 (10.84–10.97)	N.A	11.19 (11.09–11.29)	4.64 (4.61–4.68)	3.38 (3.27–3.48)
Adolescents > 12 years of age	45.19 (45.01–45.38)	N.A	58.50 (58.20–58.81)	31.62 (31.50–31.74)	8.33 (7.64–9.02)
Number of codeine prescriptions issued by a general practitioner—%	153,312 (42.06)	17,781 (33.91)	90,579 (44.69)	138,906 (39.63)	2295 (47.13)
Number of codeine prescriptions for respiratory diseases—%	103,283 (28.33)	6361 (20.96)	44,898 (22.15)	80,819 (42.41)	2449 (50.30)
Weighted mean of codeine daily dosage in mg ± SD					
Children ≤ 12 years of age	47.16 ± 14.43	N.A	67.32 ± 18.70	52.04 ± 7.92	48.55 ± 13.18
Adolescents > 12 years of age	165.08 ± 20.70	N.A	179.52 ± 27.03	187.73 ± 4.84	150.90 ± 28.10
Weighted mean of codeine cumulative dosage in mg ± SD					
Children ≤ 12 years of age	356.56 ± 64.36	N.A	457.38 ± 163.98	343.24 ± 40.36	295.65 ± 61.81
Adolescents > 12 years of age	759.87 ± 49.25	N.A	828.99 ± 72.42	678.27 ± 45.71	631.82 ± 97.75

*CI* confidence interval, ^a^Quebec included only children/adolescents born from mother covered by the Public Drug plan between 1998 and 2015.

### Impact of Health Canada safety alerts

**Figure 1:**
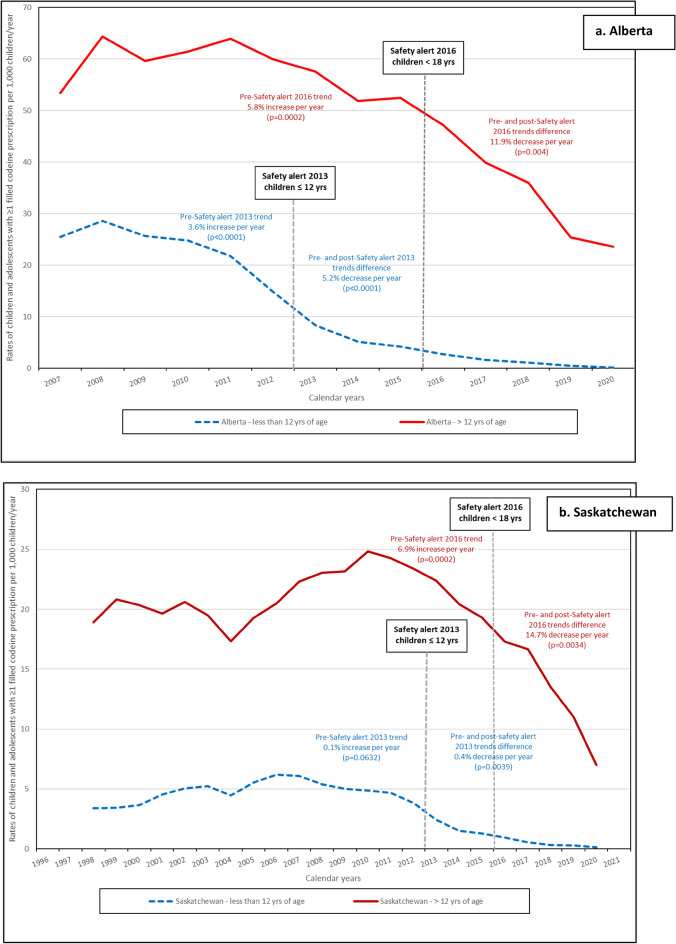

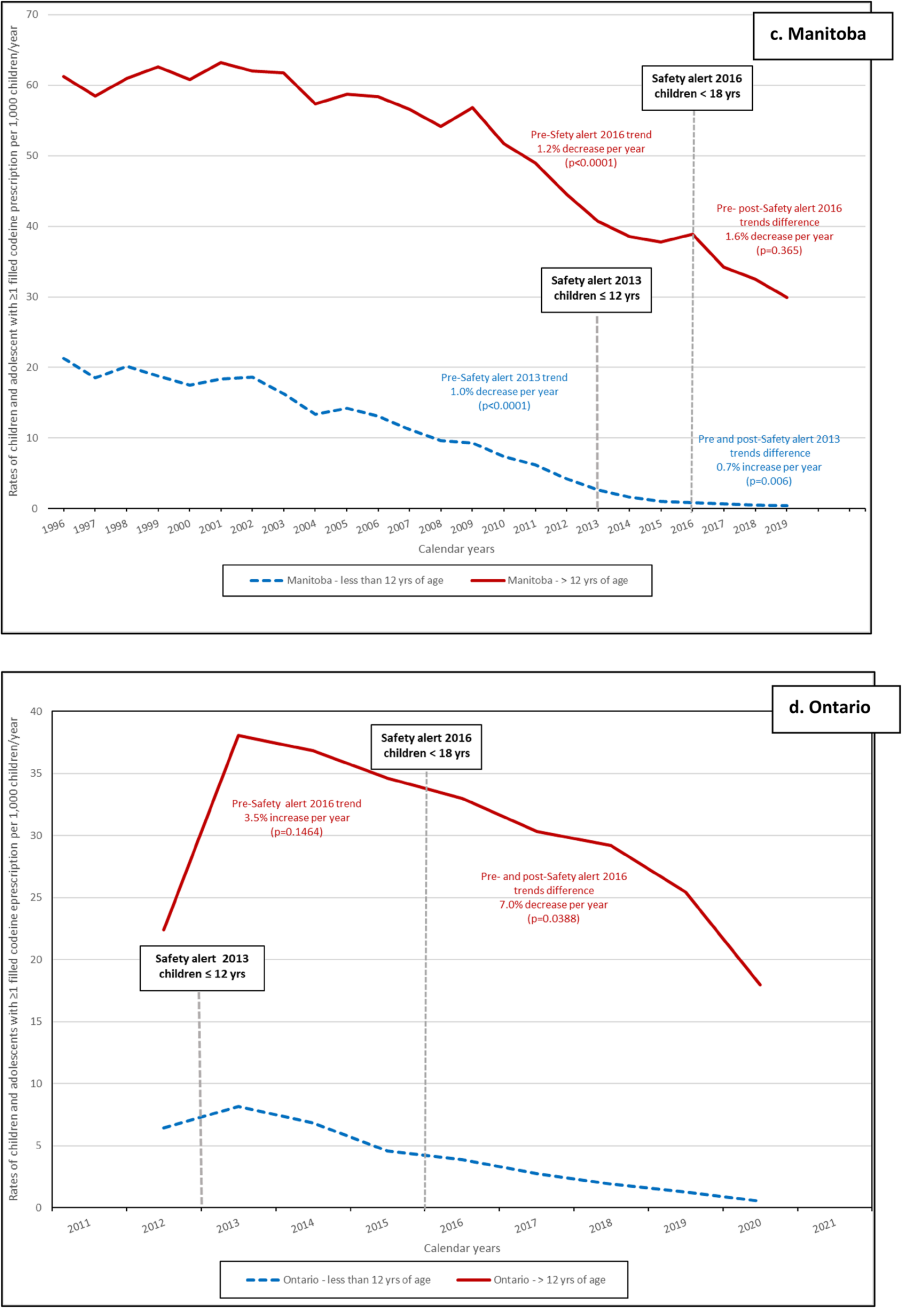

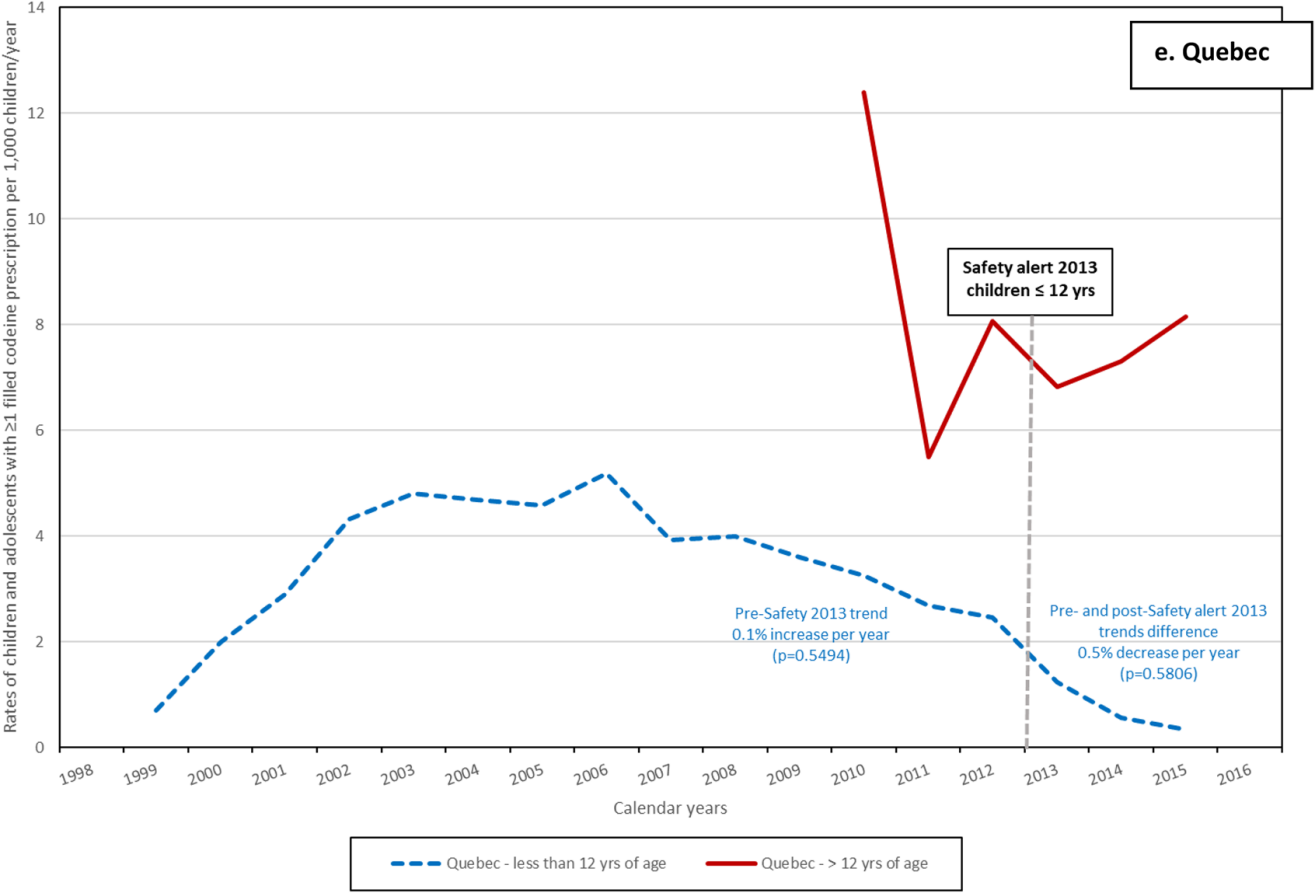
Rates of children and adolescents with ≥ 1 filled codeine prescription per 1000 children/year prior and after the two Health Canada Safety alerts. (**a**–**e**) Safety alert 2013: medications containing codeine were no longer recommended for pain or cough treatments in children (≤ 12 years of age); Safety alert 2016: codeine prescriptions were not recommended in children and adolescents (< 18 years old).

In both age categories, the rates of children exposed to codeine (per 1000 person-days) decreased during study period (Fig. 1). The impact of the HC advisory in 2013 among children ≤ 12 years old was evaluated in all provinces except Ontario, and the ITS analyses results are presented in Fig. 1 and with details in supplementary eTable 1. The difference between pre- and post-advisory trends was statistically significant in Alberta with an average annual decrease of 5.2% (*p* < 0.0001) (Fig. 1a and supplementary eTable 1); in Saskatchewan with an annual average decrease of 0.4% (*p* = 0.0039) (Fig. 1b and supplementary eTable 1); and in Manitoba with an average annual decrease of 0.7% (*p* = 0.006) (Fig. 1c and supplementary eTable 1). The 0.5% decrease per year between the 2013 pre- and post-advisory was not statistically significant in Quebec (*p* = 0.58) (Fig. 1e and supplementary eTable 1). Among adolescents (> 12) the 2016 HC advisory resulted in a statistically significant decrease in Alberta (11.9%, *p* = 0.004) (Fig. 1a and supplementary eTable 1), in Saskatchewan (2.8%, *p* < 0.0001) (Fig. 1b and supplementary eTable 1), and in Ontario (7.0%, *p* = 0.039) (Fig. 1d and supplementary eTable 1). The decrease observed in Manitoba (1.6%) was not statistically significant (Fig. 1c and supplementary eTable 1).

### Number of filled codeine prescriptions

The rate of filled codeine prescriptions among children aged 12 and below varies across provinces, with Saskatchewan having the lowest rate of 3.35 per 1000 children/years (95% confidence interval (95% CI) 3.29–3.41) and Manitoba exhibiting the highest rate at 11.52 per 1000 children/years (95% CI 11.42–11.62).Among adolescents (> 12), a higher mean rate of the number of filled codeine prescriptions were observed ranging from 8.46 per 1000 children/years (95% CI 7.77–9.16) in Quebec to 62.01 per 1000 children/years (95% CI 61.70–62.33) in Manitoba (Table 2). The comparison between the rates of the number of children exposed to codeine, the number of filled codeine prescriptions, and the treatment courses were presented by province and calendar year in supplementary eFig. 1a–d. Treatment courses were not presented for Saskatchewan where the data on medication dosage is not available for research.

### Codeine prescriber speciality and indication

About 58.9% of Canadian physicians are general practitioners, with their codeine prescriptions ranging from 33.9% in Saskatchewan to 47.1% in Quebec (Table 2). As a result, the majority of codeine prescriptions were written by specialist physicians such as pediatricians, emergency physicians, otolaryngologists, and others. (Table 2). However, the number of fillings with missing prescriber specialties varied widely between provinces, ranging from 0% in Quebec to 40.4% in Saskatchewan. The main indications (32.8%) for codeine prescriptions was respiratory diseases (Table 2). The list of indication for codeine prescriptions was presented in supplementary eTable 2.

### Mean daily and cumulative dosages of codeine

**Figure 2:**
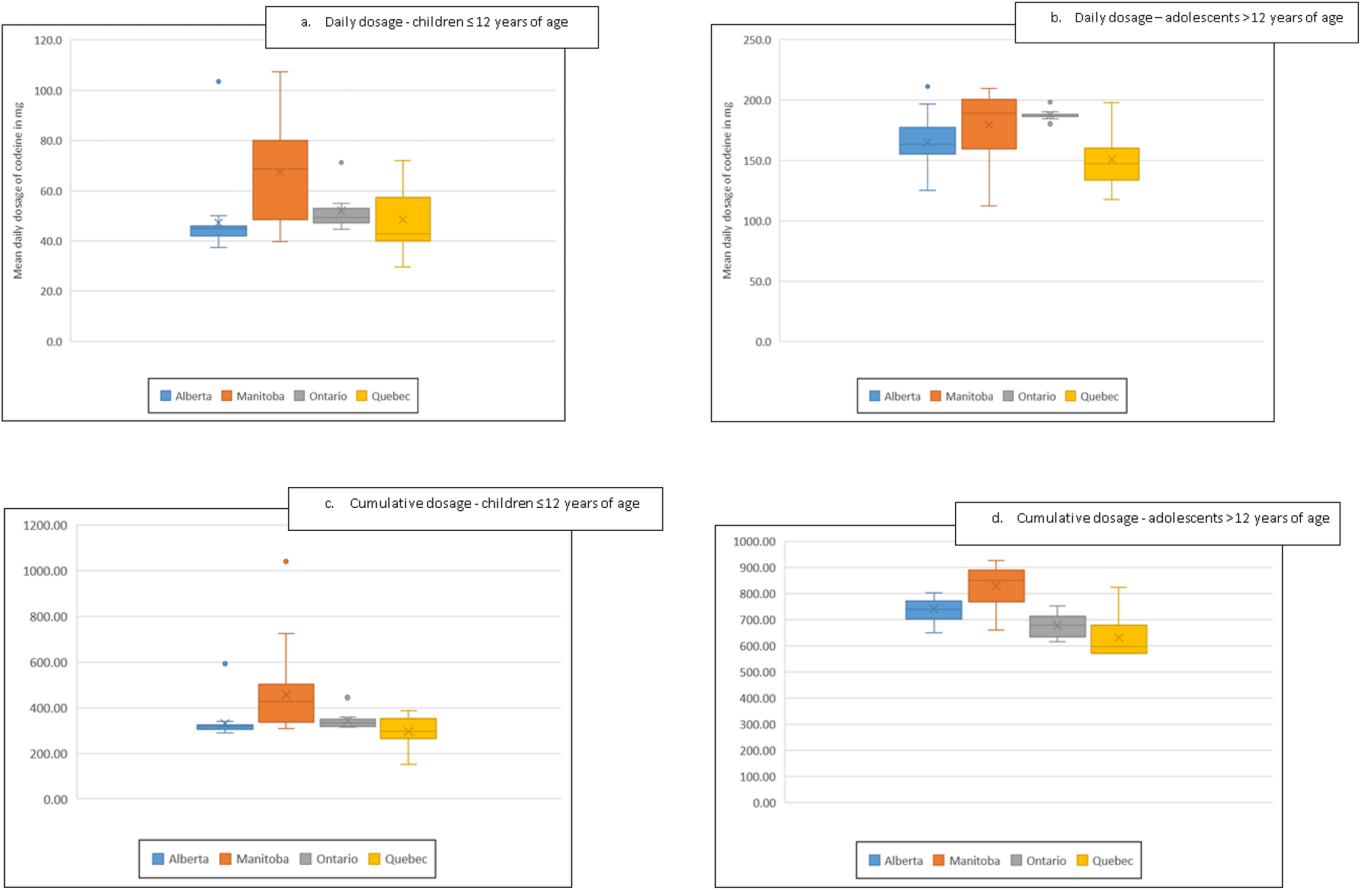
Mean daily and cumulative dosage of filled codeine prescription by province. (**a**–**d**) The distribution of mean daily and cumulative dosage are presented after removing the outlier (represented by dots). The boxes are delimited by the 25th percentile at the bottom and the 75th percentile at the top. The mean is presented by the center bar and the median by the X symbol. The horizontal lines show the minimum and maximum value.

Table 2 and Fig. 2 show the distribution of the mean for daily and cumulative dosages of codeine in mg in Alberta, Manitoba, Ontario, and Quebec; Saskatchewan did not have information on dosage. Mean daily dosage and cumulative dosage increased with increasing age categories in all four provinces. Among children ≤ 12 years of age, Alberta had the lowest mean daily dosage compared to the other provinces ranging between 47.2 mg (SD 14.4) in Alberta to 67.3 mg (SD 18.7) in Manitoba (*p* < 0.0001) (Table 2 and Fig. 2). The lowest observed mean daily dosage among adolescents was 150.9 mg (SD 28.1) mg in Quebec while the highest was 187.7 mg (SD 4.8) in Ontario (*p* < 0.0001) (Table 2, Fig. 2). However, the average cumulative codeine dosage was the lowest in both age categories in Quebec (Table1 and Fig. 2). Differences of cumulative dose were also observed between the four provinces (*p* < 0.0001 for both age categories) (Fig. 2).

## Discussion

In our study, the rate of filled codeine prescriptions decreased among children ≤ 12 years of age after the 2013 HC advisory. Trends difference between pre- and post-advisory among children ≤ 12 years of age were statistically significant in Alberta, Saskatchewan, and Manitoba. The impact of the 2013 HC advisory could not be evaluated in Ontario due to data being available only as of 2012. Higher pre- and post-advisory trend differences were observed among adolescents after the 2016 HC advisory in Alberta, Saskatchewan, and Ontario; while no differences were observed in Manitoba. The impact of this advisory could not be evaluated in Quebec as data was available only until 2015. Codeine prescriptions were most frequently written by general practitioners as was observed by Chua and Conti^11^ and were mainly for respiratory diseases. Differences in mean daily and cumulative dosage were observed between provinces. The variations of doses between provinces are probably associated with other factors, mainly different access to codeine mediations and different practices/management and these variations could be explored in another study using additional data. However, as expected, the dosages increase as children get older given that doses are often weight/age based in the pediatric population.

Codeine exposure rate estimates in our study varied greatly from one province to another by age category. However, children ≤ 12 years of age received very little codeine by the end of the province-specific study period (eFigure 2), i.e., 0.13–0.55 per thousand person-time. Observed differences between provinces could by partially explained by variations in the Canadian healthcare system. The Canada Health Act provides universal coverage hospital and medical care, however outpatient drugs are not included in this legislation^27^. Prescription drugs in Canada are covered by a combination of public and private drug plans that vary by province^28^. Two hundred twenty different Drug Identification Number (DINs) including codeine alone or in combination were available during the overall study period (1996–2021) (eTable3) and the variations on their availability in each provincial prescription formulary can explain the rate differences we observed. Indeed, variations in codeine exposure rates between provinces were also observed in three Nordic countries between 2006 and 2012 by Mahic et al*.*^29^. In a study conducted in US adolescent population aged 12–17 years by Carmona et al*.*, increase in the prevalence of opioid prescription use was observed with increasing age of children/adolescents^30^. Similar increases were also observed by Chua et al*.*^11^, Levingstone et al*.*^7^, and Bell et al*.* in Australian children/adolescents^1^ as in our study. Studies have demonstrated the decline of use of medication containing codeine during study periods and after 2013^5,6,11,25,31^. In a Norwegian study of 77,942 children and adolescents, a 90% decrease in the prevalence of opioids use was observed in children aged 0–11 years, and a 21% decrease in adolescents aged 12–14 years of age between 2010 and 2018^5^. Among adolescents 15–18 years of age, the prevalence increased by 14% between 2010 and 2012 and remained stable thereafter^5^. Chua et al*.* observed a 90.1% decrease of cough and cold medications containing codeine between 2014 and 2019 in the US^11^. The authors attribute this decrease to the FDA’s box warning on the safety of codeine use in children published in 2013^11^. In a Norwegian study from 2010 to 2018, the prevalence of filled prescriptions for children ≤ 11 years of age declined significantly between 2013 and 2014, suggesting that FDA warnings may have effects outside the US (since other countries may adopt similar strategies for a health threat addressed by the FDA)^5^. Decreasing rates of filled codeine prescription observed in our study are similar to those obtained in the other studies^4,5,25,31^, although the comparison between our results with others is limited by the study period.

Our study has several strengths. First, we included five population-based, retrospective cohorts with linkage of medical and pharmaceutical data at the individual child/adolescent level. Second, the pharmaceutical data include detailed information regarding codeine exposure such as date of filling, quantity of drug provided, duration of the prescription, dosage, and formulation. The date of the filling allowed us to calculate the timing of codeine prescription dispensation and the other variables (e.g. mean and cumulative dose). We also used prospectively and routinely collected physician-based diagnoses for the identification of the potential indication for the codeine prescription. Prescription databases are a good source of valid and reliable data to study drug use^32^.

Despite these strengths, our study has some limitations. Our assessment of codeine exposure relied on filled prescriptions rather than actual intake. However, Zhao et al.^33^ demonstrated that prescription filling is a valid measure when compare to actual use. Differences in duration and timing of the study period per cohort limited the evaluation of the impact of HC safety alerts for both age groups in all provinces. Additionally, the fact that Quebec included only liveborn of mothers who were covered by the provincial public drug insurance could affect the rate comparability with other provinces. Differences in the number of medications containing codeine between provinces are explained by the variation in reimbursement formularies and provinces with a greater number of medications on its formulary has a greater chance of having a higher exposure rate. Although HC and pediatricians recommend against their use, several cough syrups containing codeine are available over the counter, and as such the true rate of codeine exposure is underestimated.

The publication of codeine safety alerts by HC has demonstrated a decrease in the number of children and adolescents exposed to medications containing codeine across all five provinces included in this study. Among children aged 12 and under, the largest decrease of the adjusted-trend after the 2013 safety alert was observed in Alberta, with a decrease of 5.2% (*p* < 0.0001). For adolescents over 12 years of age, the most substantial impact of the 2016 safety alert was observed in Saskatchewan, with a decrease of 14.7% (*p* = 0.003). This emphasizes the importance of safety alerts issued by regulatory agencies. These data are also important for clinical practice as well as for decision makers such as HC.

## Methods

### Study design

This study used data from the Canadian Mother and Child Cohort (CAMCCO) active surveillance initiative. The CAMCCO data are not publicly available but variables included in each province mother and child cohort are described in Maelstrom Research^34^ and has been presented in detail in Bérard et al.^35^. In short, CAMCCO is a population-based cohort, which includes administrative health care and hospitalisation data from five Canadian provinces: Alberta, Saskatchewan, Manitoba, Ontario, and Quebec. Each provincial children/adolescents cohort includes prospectively collected harmonized data on medical services, filled drug prescriptions, and hospitalizations. Medical service databases include information on all primary care services, including physician-based diagnosis (International Classification of Disease 9th and 10th revisions (ICD-9, ICD-10)), health care providers’ unique identifier and speciality, medical procedures, and calendar dates of when medical procedures occurred. Prescription drug databases, except for Ontario, include information on all filled prescription medications, the prescribing clinician’s specialty, and the pharmacist’s unique identifier, prescription start date and duration, drug identification number (DIN), dosage, and quantity. Hospitalization databases provide information on diagnostic codes (ICD-9, ICD-10), interventions, procedures, and consultations. For Ontario, data were obtained through the Institute for Clinical Evaluative Sciences (ICES). Physician speciality and potential indications for codeine prescriptions was obtained from the ICES physicians’ database. Prescription information was obtained from the Narcotics Monitoring System (NMS) where dispensers are required to submit dispensing information about all monitored drugs including narcotic analgesics. Finally, through a unique patient encrypted identifier, databases in each province were linked together and patients were followed over time.

### Setting

The analyses were performed in each province using a common, pre-specified protocol (Supplementary eMethods 1). Each province had access to different study period lengths with the longest being in Manitoba (1996–2019) and the shortest in Ontario (2012–2020) which are presented in Supplementary eFig. 2. Due to legal constraints, individual data cannot be shared. Analysts in each province perform analyses in accordance to a common protocol and standardized definitions for identifying exposures, outcomes, and co-variables. The lead analyst generates SAS programming codes, which are shared among the provinces. The analysis results (aggregated data) are subsequently transferred to a secure cloud space using previous provided shell tables.

### Participants

For each calendar year during each provincial study period, all children/adolescents between 0 and 18 years of age were identified using the province-specific demographic database and classified into age groups (≤ 12 and > 12 years of age). The study entry date was the child’s date of birth in Quebec. Children/adolescents aged 0–18 years could enter at any time during the study period in the remaining four provinces. Each child/adolescent was followed until their death, 18th birthday, end of drug insurance plan coverage, or the end of the provincial study period, whichever came first. A child/adolescent was classified in both age categories during the calendar year of their 12th birthday. The number of potential exposure days per calendar year was calculated for each participant and assigned to the appropriate age category.

### Exposure

Using provincial prescription drug databases, we retrieved all filled codeine prescriptions per calendar year, classified by age category. In Canada, coverage for prescription drugs exists through an array of public and private drug plans, however there is no requirement for provinces to provide outpatient medication coverage to all residents^36^. Indeed, a product approval for marketing by HC does not automatically ensure its’ public reimbursement across Canada. The decision to list a drug is under provincial jurisdiction leading to differences between provincial drug reimbursements formularies. Codeine exposure was defined dichotomously as any prescription filled for codeine alone or in combination with other medications. We included all codeine prescriptions with oral administration (syrup/elixir/liquid/solution/suspension or tablet/capsule). Identification of filled codeine prescriptions was done using the DIN present in the pharmaceutical dataset of each Canadian province (Supplementary eTable 3). For each calendar year and the two age groups, we first identified the number of participants who filled at least one codeine prescription. The annual rate was calculated by dividing the number of users by the total number of potential exposure days for each calendar year and age category (supplementary eFig. 3). We also calculated the number of filled codeine prescriptions per participant. Finally, we determined the number of codeine treatment courses accounting for a grace period between prescriptions equal to half of the duration of the first prescription (Supplementary eFig. 4). The daily and cumulative dosage of codeine prescriptions were calculated in each province except for Saskatchewan where medication dosage data was not available.

### Other variables

The prescribing clinician’s specialty was identified for each filled codeine prescription and classified into one of the following categories: general practitioner, pediatrics, general surgery, otolaryngology, urology, or other. Within 10 days before the first day of the codeine filling, we used the medical service databases to identify the more recent medical visit with the prescriber to define the potential indication for codeine use. ICD-9 diagnostic codes were used to classify the indication for each filled codeine prescription (Supplementary eTable 2).

### Statistical analysis

The number of children/adolescents included in each provincial cohort was determined using unique child/adolescent encrypted identifier and the weighted mean years of follow-up was calculated using the total number of children/adolescents in each province as a weight. In each province for each calendar year, the rate of children exposed to codeine per 1000 person-days was obtained using the ratio of the number of children/adolescents with at least one filled codeine prescription over the number of person-days contributed by all children/adolescents in the province in that calendar year, multiplied by 1000 (supplementary eFig. 3). Provincial rates were calculated for both age categories. The rates of the number of codeine prescriptions and the treatment course were obtained using the same calculation (supplementary eFig. 4). We used interrupted time-series (ITS) analysis with segmented regression analyses to model changes in the levels and trends of codeine use associated with the following time points: 2013 HC advisory (≤ 12 years of age) and 2016 HC advisory (> 12 years of age). The level changes in the year following each time point were controlled for the trends prior to HC safety alerts. Proportions were used to compare the indication for codeine use and the speciality of the prescribing clinician between provinces. Means with 95% confidence intervals (95% CI) were used to present the daily and cumulative dosages for filled codeine prescriptions by age category in each province. We used analyses of variance (ANOVA) to assess provincial differences of daily and cumulative dosages. Statistical analyses were conducted using SAS v.9.4 (SAS Institute Inc.).

### Ethics approval

Ontario used data from Institute for Clinical Evaluative Sciences (ICES) and the project was reviewed by ICES’ Privacy Office and audited/overseen by Sunnybrook Health Sciences REB. The other provinces received approval from their ethics committees (Alberta – Ethic protocol numbers Pro00114981 and Pro00114982; Saskatchewan received authorization from the Biomedical Research Ethics Board (Bio-REB) of the University of Saskatchewan (application id: 2094); Manitoba – approval HS24269 (H2020:412) and HIPC: 2020/2021-71, Quebec received the approval from the Ethics Review Board of Centre Hospitalier Universaire Sainte-Justine, Montreal (reference number: 1740 and 2976) and the Commission d’accès à l’information authorized the linkage between databases (reference number: 1005446-S)The study was performed in accordance with Canadian research regulations. The study was conducted using administrative data administered by the provincial health systems of Canada. All data were anonymized to preserve the confidentiality of individuals. No individual information can be shared between provinces, and only aggregated data resulting from analyses has been shared. Therefore, no consent needs to be obtained from the individuals included in our study.

### Supplementary Information

Supplementary Information.

## Data Availability

The data from the Canadian Mother–Child Cohort (CAMCCO) that support the findings of this study cannot be shared openly and only aggregate data could be shared between Canadian provinces. Details of the data included in the CAMCCO are available in the Maelstrom Research web page at: https://www.maelstrom-research.org/study/camcco
