# Supplementary material for: Health Canada advisory impacts on the prevalence of oral codeine use in the Pediatric Canadian population: comparative study across provinces
Source: Sci Rep. 2024 Mar 4;14:5370. doi: 10.1038/s41598-024-55758-3 (PMC10912710; doi:10.1038/s41598-024-55758-3)
Supplement: Supplementary file 1 — Supplementary Information. [file 41598_2024_55758_MOESM1_ESM.docx]

**Supplementary Online Content**

Sheehy O, Eltonsy S, Hawken S, Walker M, Kaul P, Winquist B, Savu A,

Dragan R, Pugliese M, Bernatsky S, Bérard A

**Health Canada advisory impacts on the prevalence of oral codeine use in the Pediatric Canadian population: Comparative study across provinces.**

.

eMethods 1. Common, pre-specified protocol

eTable 1. Interrupted Time-Series (ITS) analyse results.

eTable 2. List of indications for the codeine fillings with ICD-9 diagnosis codes (3 digits)

eTable 3. List of DIN numbers of the study medications

eFigure 1. Number of children with codeine exposure, number of codeine prescription fillings, and number of treatment courses among the pediatric population by age category, calendar year and Canadian provinces

eFigure 2. Study periods and total children included in each province

eFigure 3. Example of prevalence of codeine exposure and person/days contribution in 2010 for the children #01.

eFigure 4. Example of prevalence of codeine exposure and person/days contribution in 2010 for the children #01.

This supplementary material has been provided by the authors to give readers additional

information about their work

**eMethods 1.** Common, pre-specified protocol

**DATE:** June 7, 2019

**Team name:** CAN-AIM and the Canadian Mother-Child Cohort (multi-DSEN Teams: CAN-AIM, CNODES, and experts in the Canadian Mother-Child Cohort)

**Leader:** A Bérard (QC)

**Co-investigators**: S Bernatsky (QC), C Moura (QC), M Walker (ON), S Eltonsy (MB), D Chateau (MB), B Windquist (SK), P Kaul (AB).

**Query:** Q19-06 - Cough and cold products containing opioids: prescribing and patient utilization patterns in the pediatric population.

**A.1 Research Question**

**What are the physician’s prescribing patterns and patients’ use patterns of prescribed cough and cold opioid-containing medicines (CCOMs) in paediatrics?**

This proposal will be performed using data from the Canadian Mother-Child Cohort Active Surveillance Initiative (DSEN/CIHR/HC; Bérard et al.). In addition to overall province-specific prevalence and annual prevalence of CCOMs prescribed (physician) and filled (patient) in the pediatric population, prevalence will be calculated stratifying on age categories (infant, toddler, child, teenager, ≤12 yr old (contraindicated according to Canadian guidelines), >12 yrs old), on CCOM’s active ingredient, and on specific CCOM pattern of use.

In order to assess physician’s prescribing patterns and patient utilization patterns the following issues will be considered:

**For physician prescribing practices:** conditions for the treatment with CCOMs, prescription refills, and physician specialty.

**For patients’ utilization of CCOMs:** specific CCOM(s) filled, duration of treatment, changes in dosage, duration.

**A.2 Rational**

Opioid-related harms constitute a major public health concern in Canada, currently ranked as second in per capita prescription opioid use, after the United States. The last Canadian national report of apparent opioid-related deaths, updated in December 2018, revealed that there were more than 9,000 apparent opioid-related deaths between January 2016 and June 2018. Of them, 2,066 occurred between January and June 2018 and 94% were accidental. Most accidental apparent opioid-related deaths involved fentanyl and fentanyl analogs and occurred among males (76%), and middle age adults. Only 1% of accidental apparent opioid-related deaths occurred in <19 years of age^^[[1]](#footnote-1)^^. There are no data that discuss the role of CCOMs in the opioid related harms occurred in Canada.

There are approximately 29 cough and cold prescription products containing codeine, hydrocodone or normethadone available in Canada. Although it is known that these products, as opioids, may be misused and abused, no current data are available to quantify the prevalence/incidence of abuse, misuse, addiction, overdose and dependence in children associated with opioids in different formulations^^[[2]](#footnote-2)^^.

In 2017, the FDA undertook a comprehensive benefit-risk assessment on the use of prescription CCOMs in children, concluding that due to serious safety issues e.g., respiratory depression, death, abuse, misuse and overdose associated with these products their harms outweighed their potential benefits in the pediatric population for treating cough and cold. The FDA officially removed that indication for CCOMs on January 11, 2018. Health Canada conducted a signal assessment (SA)^[[3]](#footnote-3)^ regarding the risk of abuse, misuse and OUD in children with the use of CCOMs (review completed in September 2018). The SA identified a knowledge gap in the available evidence regarding a potential association between abuse, misuse, OUDs, and CCOMs in the Canadian pediatric population. Based on the SA, Health Canada advised that Canadian children and adolescents (under 18 years of age) should not use cough and cold products containing codeine, hydrocodone and normethadone, as a precautionary measure^[[4]](#footnote-4)^.

Given the identified knowledge gap, studies aiming to address the potential role of CCOMs in the opioid-related risks among the pediatric population, should help to provide Canadian prescribers and patients with meaningful information for decision making, validate the recommendation provided in the SA and help to understand the individual contribution of CCOMs to the National opioid crisis.

**A.3 Data Sources**

To date, the majority of studies on the risks and benefits of medication use during childhood include small sample sizes, lack of statistical power, or have sub-optimal study designs to investigate rare outcomes. To circumvent these limitations, in recent years, large national administrative databases or registries have been increasingly used in the field of perinatal/paediatric pharmacoepidemiology, recognizing the importance of large size longitudinal pregnancy and child cohorts.

We propose to bring together data from the Canadian Mother-Child Cohort Active Surveillance Initiative funded by DSEN/HC, which includes data from five provinces - Quebec (QC), Ontario (ON), Manitoba (MB), Saskatchewan (SK), Alberta (AB) to answer this query.

The ***five provincial birth cohorts*** that we intend to align will be developed by linking several health care administrative databases available through the individual provinces’ ministries of health. Healthcare administrative databases have become a cornerstone in the process of assessing performance and providing feedback to improve quality of health care delivery and outcomes at a population-level. The single-payer integrated system of health care delivery in Canada has facilitated the linking of inpatient, outpatient (including emergency department (ED) visits), hospital, ambulatory care, pharmaceutical, immunization, and laboratory data in the six provinces; the linkage between administrative databases and chart data or registries is also possible and provides further replicability potentials.

**Databases *already* available to researchers in each province (Table 1.):** The variables available in all provinces are presented in Table 1. Briefly, the medical service databases (Medical Services file) contain detailed information on all medical services, including physician-based diagnosis and therapeutic procedures, diagnoses coded according to the International Classification of Diseases, ninth and tenth revisions (ICD-9, ICD-10), as well as the date and information on the institutions where the medical procedures were performed. Health care provider characteristics are also included. The Prescription Drug file covers information on all filled prescribed medications, the prescribing physician and dispensing pharmacist, drug name, dosage, formulation, quantity dispensed, date and duration of the dispensation for publicly insured people. In the QPC, data in the Prescription Drug file have been validated and found to be highly reliable *(Zhao et al., 2017, BDRA).*

The hospitalization databases (archives databases) record all acute care hospitalization, including the length of gestation. Data on physician-based medical diagnoses are also available in these databases.

Birth and death databases provide demographic information on the mother, father, and baby as well as birth weight and gestational age for live births and stillbirths. Data recorded in the QPC have been compared to medical charts and found to be complete and valid *(Vilain et al. 2008 PDS)*.

In order to create the province-specific cohorts, the linkage between the databases will be performed using a patient unique encrypted identifier. Each subject’s unique encrypted identifier will be provided to the research team by the Medical Service database providers. Children will be followed from birth until the end of 2018.

*Table 1. Description of the variables, calendar years, sample sizes, and aggregate data to download in our secure repository in Montreal, CHU Ste-Justine.*

| **Field Name** | **Dictionary** | **Description** | **Administrative databases within each province** | **Canadian Mother-Child Cohort  (Aggregate Data)** |
| --- | --- | --- | --- | --- |
| **Province – Where linkage will be done** |  |  | QC, ON, MB, SK, AB |  |
| **Brief Database Description** | Description of database including origins, unique characteristics, general or specific users, as well as strengths and limitations of the database | One Paragraph Description of Database | Each province-specific Mother-Child Cohort will be built by linking administrative hospital and sociodemographic databases. It will contain data on mothers and children at birth (medical charts, lifestyle data, family and personal medical history) and data on pregnant women and their children from 1997 to 2017. It is planned that we will update the cohort data annually (data on mothers, children, new pregnancies). These 6 Mother-Child provincial cohorts will have the advantage of large size compared to the other data sources whose sample sizes are often insufficient to rule out low-to-moderate increased risks for event the more commonly occurring adverse perinatal outcomes such as heart defects, neural tube defects, and oral clefts. All linkages will be done by denominalized unique identifiers for mothers/children; the mother-child link is ensured by a unique mother-baby link already available in each province. This is NOVEL given that it has never been used before in Canada and elsewhere. | Aggregate data (population means, prevalences, risk ratios/odds ratios/hazard ratios on all important/relevant variables) will be calculated within each province, using provincial-based Mother-Child Cohorts and will be centralized within a unique repository in QC (secure server, CHU Ste-Justine). **This model has been used before in the US FDA Sentinel Program. It gives us the opportunity to answer policy relevant questions without reanalyzing all the individual cohorts.** In the event that more refined measures of risk are needed, a common protocol will be developed and analyses within each province will be done and results meta-analyzed. |
| **Database Type** | Description of type and scope of data: Population Database | **I. Longitudinal Population Database**  **A.** **Drug and Diagnosis Data**  Medical and Pharmacy  Insurance Claims –   *outpatient and inpatient*  **B. Diagnosis Data only**   1. Medical Claims  2. Electronic Records   3. Disease Specific  **C. Drug Data only**  Pharmacy-based –  *medications fillings,  outpatient*  **II. Spontaneous Reporting System**   **A. Standard   B. Usual Universal Care** | Longitudinal Population Database |  |
| **Database Source** | Data origin: e.g., medical insurance and medication filling claims, hospital archives, demographic databases (births and deaths) |  | **Medical Insurance Claims:** Physician visits, diagnoses with calendar date (ICD9-10 codes), procedure codes, medication fillings (calendar date, name of drug (DIN), dosage, duration), sociodemographic data on prescribers, visits to emergency).  **Hospitalization database (hospital archives):** Diagnoses during hospitalization (calendar date), gestational age, procedures, data on delivery or end of pregnancy (spontaneous or planned abortion). **Birth and Death certificate data:** Data on child (gestational age at birth, birth weight), marital status of mother, education level, race/ethnicity. | Aggregate data from all 6 provinces:  Population means, prevalences, risk rations/odds ratios/hazard ratios, etc. on all important/relevant variables. |
| **Frequency of Data Collection** | How often data is collected, such as ongoing, annually or quarterly |  | Ongoing | All aggregated data produced as studies will be performed in the province-specific cohorts will be downloaded in the General Canadian Mother-Child Cohort. |
| **Frequency of Data Update** | How often the database is updated |  | Quarterly |  |
| **Years Covered** | Which period the database covers |  | 1997-2017 | 1997-2017 |
| **Population Type** | Description of population type (by demographic, insurance status, region) | General Population  Outpatient/Non-institutionalized  Inpatients  Emergency room (ER/ED)  Neonates | General Population  Outpatient/Non-institutionalized  Inpatients  **Note:** QC only covers welfare recipients or adherents for their medications; ON only covers welfare recipients and families with children for their medications; BC, AB, DK, MB cover all their population for their medication. All other health visits and procedures are universally covered. | All aggregated data produced as studies will be performed in the province-specific cohorts will be downloaded in the General Canadian Mother-Child Cohort. |
| **Date of Last Update** | When the database was last updated |  | The QPC has been developed for the period 1997-2015 for mothers/children covered by the RAMQ for their medications (30% of the overall population) – we are asking to obtain data on all pregnancies/children in QC from 1197-2017 regardless of medication insurance status within this proposal. All the other 5 provinces never had a Mother-Child cohort and we are therefore asking for funding to build the 5 infrastructures within this proposal. |  |

| **Size** |  |  | QC: At present – 500,000 pregnancies and 400,000 children followed for up to 18 years (1997-2017) – it is estimated that with the additional data requests (data on ALL pregnancies and children in QC from 1997-2017 – 1 million pregnancies, 800,000 children. ON – it is estimated that we will have data on 500,000 pregnancies and 400,000 children. MB, SK, AB - 500,000 pregnancies and 400,000 children for each province. BC – 700,000 pregnancies and 550,000 children. Overall, approximately 3.4 million pregnancies and 2.95 million children. |  |  |
| --- | --- | --- | --- | --- | --- |
| **BORN (ONTARIO)** | | | | | |
| -BORN Ontario is a prescribed registry under Ontario PHIPA (Personal Health Information Protection Act), which collects, discloses, and uses personal health information for the purpose of improving care and patient outcomes. The BIS, an Internet-based data collection system, is operational in all 96 hospitals providing maternal-newborn care and has data for all hospital births since 2006. Maternal demographics and health behaviours, pre-existing maternal health problems, obstetric complications, intrapartum interventions, and maternal and newborn outcomes are captured at the time of birth. Each site has access to their own data and BORN Ontario reports on outcomes aggregated at the provincial level at regular intervals. An ongoing data validation process assures high data quality, and a number of studies using BORN data have been published. With comprehensive capture of high-quality data for all births in the province, BORN developed reporting tools to alert hospitals to potential practice issues to facilitate practice improvement. | | | | | |

**A.4 Methodology**

Within each provincial administrative/hospital databases, a birth cohort will be formed including all births that occurred in the province between 1998-2018. Date of entry in the cohort will be the date of birth (DOB) and children will be required to be publicly insured for their medications.

Follow-up will be done from DOB until i) the child is 18 years of age, ii) end of medication coverage, or iii) December 31, 2018, whichever comes first.

CCOMs considered will be fentanyl, codeine, hydrocodone, normethadone, dextroethorphane alone or in combination forms (i.e. acetaminophen and codeine, etc.); pills, syrups, oral formulations or elixirs will be studied.

- CCOM use will first be defined dichotomously (y/n) on an annual basis, within each province (Annual province-specific prevalence) to estimate pattern of use.
- Beginning of use will be defined as the first day of the first prescription filling after DOB.
- Duration of use **per treatment course** will be defined in 2 ways (commonly used definitions for acute medication exposure in drug utilization studies):

1. From the first day of the first filling until the last day of the last consecutive fillings (i.e. the first filling was for 10 days, and the child has a refill on day 11 for another 10 days – the child is considered exposed for 20 days).
2. Given that these prescriptions are used on a as-needed basis and not necessarily as prescribed, we will allow for a grace period in between fillings where we will consider the child continuously exposed. This grace period will be 50% of the previous filling (i.e., if a filling is for 10 days, we will allow the next filling to be done within day 11 to day 15 (1 to 5 days after the end of the previous filling) and will consider the child exposed for up to 5 days in-between fillings. If however, the second filling is done after the 50% grace period, the child will be considered exposed for only the 10 days (treatment course 1); the second filling will be considered a second course of treatment.

Both 1) and 2) will be used to determine the number of refills overall, and by treatment courses.

- Treatment course: Treatment course will be defined as the number of treatments without overlapping durations (see above, 2)) from DOB until the end of follow-up (see above).

1. Annual prevalence: from 1998 to 2018, annual prevalence of CCOMs will be calculated within each provincial birth cohorts, and an overall CCOMs prevalence of use will be calculated as a combined measure for the 5 participating provinces. This will be calculated as 1) overall CCOMs, 2) CCOM types (codeine, hydrocodone, normethadone, dextroethorphane alone or in combination forms), and 3) CCOM intake forms (pills, syrups, oral formulations or elixirs). Furthermore, stratification will be done by age categories: i) infant (0-12 months), ii) toddler (1-3 years of age), iii) child (3-12 years of age), iv) teenager (13-18), and v) ≤12 years of age vs. >12 years of age (this will be used to define ‘misuse’ given that opioid related combinations are contra-indicated for those ≤12 years of age).
2. Age (from DOB) at the first prescription filled for CCOMs will also be measured.
3. For each medication filling, we have the specialty of the prescribing physician. This will be used to calculate the frequency of prescribing among each of those who did prescribe a filled study medication. Stratification on children’s age will also be done here given the guidelines not to prescribe to those ≤12 years of age.
4. For each prescription filling, we will look 10 days before and after the first day of the filling to identify the potential indication for use (condition to be treated) using ICD9/10 related codes for cough, pain, and fever. From the feasibility assessment within the QPC (see below), the majority of fillings are for cough, pain, and fever. Although this will likely give us a valid measure of the reasons why CCOMs are prescribed, we will validate these diagnoses identified in the last population-based province-specific cohorts using patient charts data from the QC Project 1-2-3 (patients charts from a representative sample of the QPC), and ON BORN (Child cohort – patient charts).
5. Average daily dosage and cumulative dosages of fentanyl, codeine, hydrocodone, normethadone, dextroethorphane will be calculated using morphine equivalence dosing during each treatment courses.
6. Change in dosage will also be calculated within each course of treatment using morphine equivalent dosages for each of the studied CCOM. This gives a way to make all drug-specific prescribed doses equivalent in terms of potency and thus study dosages and dosage changes taking into all CCOMs together.

Comparisons between provinces, by calendar year, and age groups will be done across all 5 participating provinces.

Crude data will remain in each province but aggregate data from each participating province will be downloaded in the Canadian Mother-Child Active Surveillance Maelstrom website (<https://www.maelstrom-research.org/>) - ongoing.

**A.5 Strengths and weaknesses**

Strengths – Large province-specific birth cohorts will be built to study CCOMs in the pediatric populations. Medication reimbursement program differences between provinces will allow the comparisons of findings between provinces, and thus will give a clearer picture of the overall use of CCMOs in this specific population. Using standardized protocols and procedures will result in higher validity. Limitations – We will have data on fillings of prescribed CCOMs and will not include OTC CCOMs that are obtained without a prescription. This should not be a clear limitation for this query given that it is focused on prescribing patterns of CCOMs. Fentanyl use will likely not be fully captured here given that it is not often prescribed (although from experience, we have prescribed Fentanyl in these study databases (Berthod et al., Prescribed opioids during pregnancy – a Drug utilization study within the Quebec Pregnancy Cohort. AJOG, submitted). Again that will result in ‘prescribed’ fentanyl, which is in direct line with the query.

This proposed study will give the change to compare CCMOs use in children in Canada. It is also important to compare our findings to other countries. Hence, to complement our study and have an international comparison, we will reproduce our analysis in two US databases: MarketScan® Commercial Claims and Encounters (Commercial) database and Medicaid Multi-State database. Marketscan Commercial database that contains individual-level healthcare claims for millions of privately insured patients with many different health plans from large employers, public organizations, and government. Medicaid database contains claims from Medicaid enrollees from eleven geographically dispersed states. It includes long-term care claims file that captures nursing home stays, home health care, and long-term care services.

1. **Please include an estimate for the project duration / timeline and a rough estimation of the resources ($) required above and beyond those covered by your team grant**

Duration – 12 months

Budget 200,000$ - 37,500 $/participating province for data access, and analyst. 12,500$ additional for the Qc group for the coordination, upload of results into Maelstrom, and analyses to combine all province-specific results into common, combined Canadian indicators.

1. **Supplementary information needed from the questioner:**

This study is highly feasible. We have the buy-in of all 5 participating provinces.

Although we have not been able to interrogate all 5 provincial databases, we were able to identify in QC (QPC) 6,292 CCMOs filled between 1998-2015, for 2,968 children; 227 Rx for syrups with codeine, 3,496 for syrups with acetaminophen and codeine, 1,137 Rx oral codeine, 1,125 Rx oral acetaminophen with codeine, and 1 Rx syrup with dextroethorphane (contraindicated). The majority of these prescriptions were for children <= 12 years old (contraindicated) (4,484 Rx).

In ON, 26,821 people aged <=18 received one of these products in 2016.

1. **Feasibility by your methodology (please comment)**

Unfeasible  Feasible with modifications

Partly feasible  Feasible

**eTable 1.** Interrupted Time-Series (ITS) analyse results

|  | **Safety alert 2013** | | | | **Safety alert 2016** | | | |
| --- | --- | --- | --- | --- | --- | --- | --- | --- |
|  | **Children ≤ 12 years of age** | | | | **Children > 12 years of age** | | | |
| **Province** | **Level change^1^** | **p-value** | **Difference^2^** | **p-value** | **Level change** | **p-value** | **Difference** | **p-value** |
| Alberta | - 18.4% | 0.0014 | -5.2% | <0.0001 | -15.2% | 0.3176 | -11.9% | 0.0038 |
| Saskatchewan | -3.0% | 0.0002 | -0.4% | 0.0039 | -1.7% | 0.40 | -2.8 | <0.0001 |
| Manitoba | -3.4% | 0.0048 | -0.7% | 0.006 | -2.1% | 0.686 | -1.6 | 0.365 |
| Ontario | N.A. | N.A. | N.A. | N.A. | -0.7% | 0.920 | -7.0% | 0.039 |
| Quebec | -2.2% | 0.286 | -0.5% | 0.581 | N.A. | N.A. | N.A. | N.A. |

^1^Level change following safety alert after controlling for the trend prior safety alert. ^2^Difference between pre- and post-safety alert trend.

**eFigure 1.** Number of children with codeine exposure, number of filled codeine prescription, and number of treatment courses among the pediatric population by age category, calendar year and Canadian provinces

1. **Alberta**


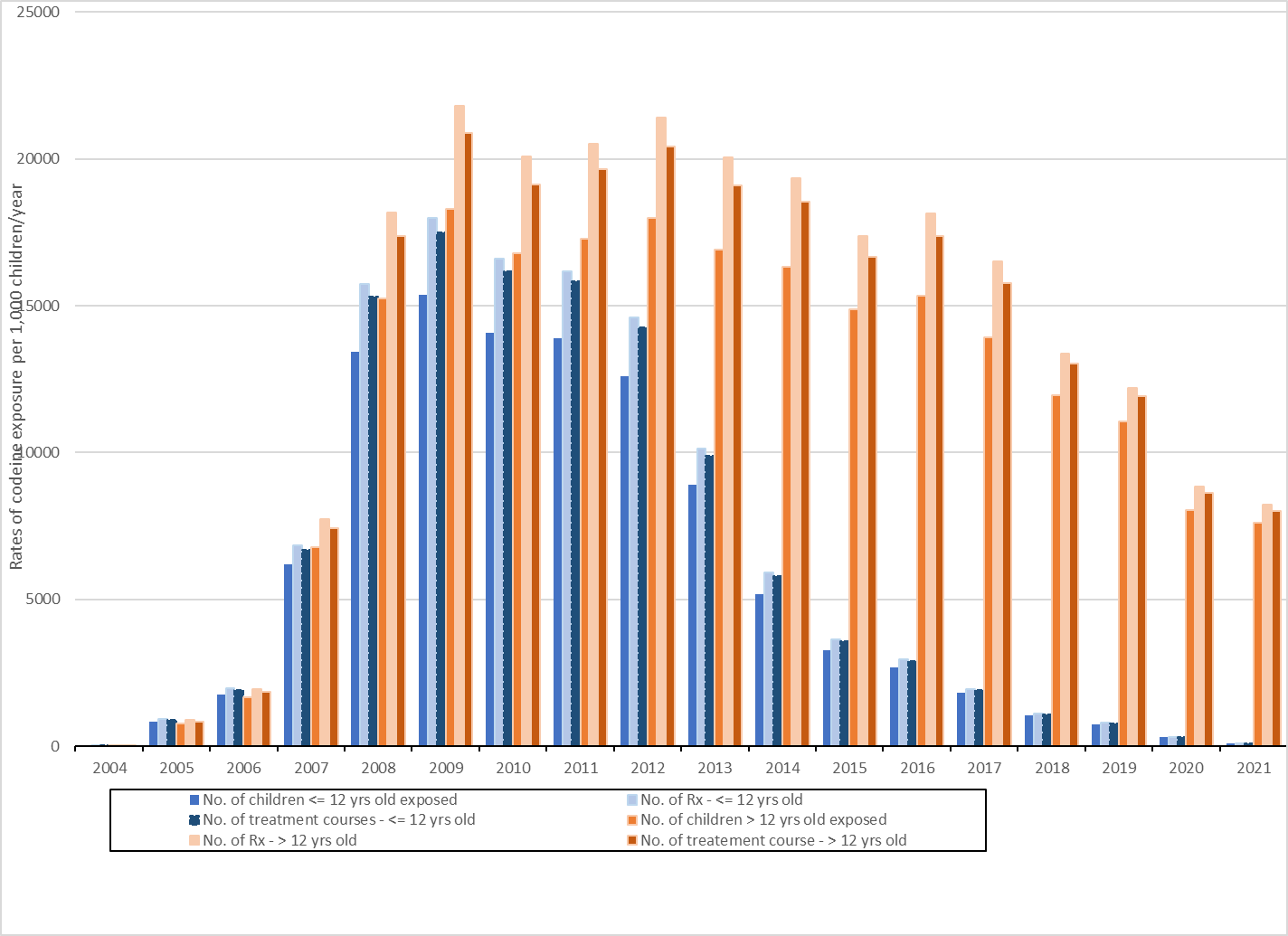


1. **Manitoba**


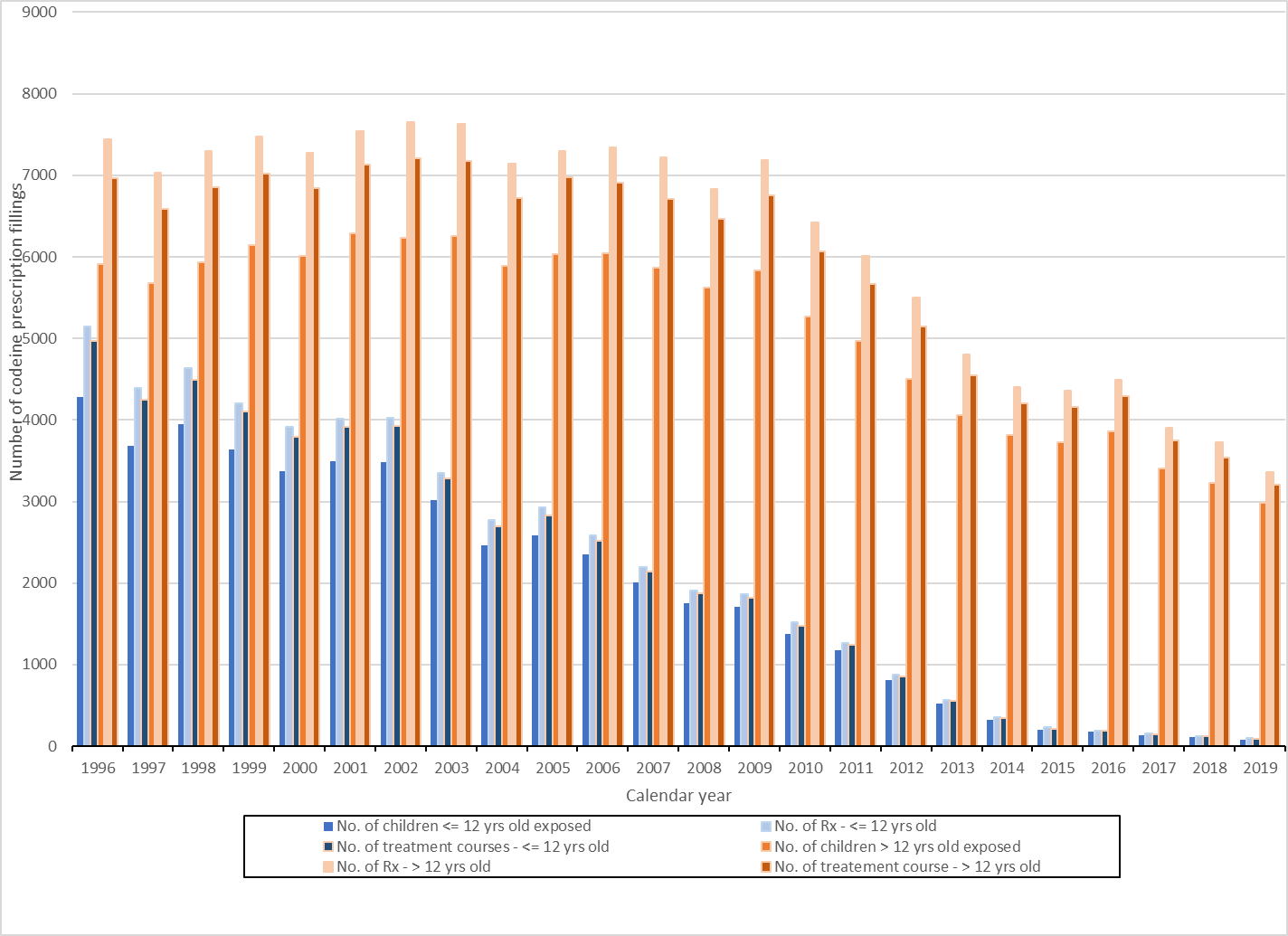


**
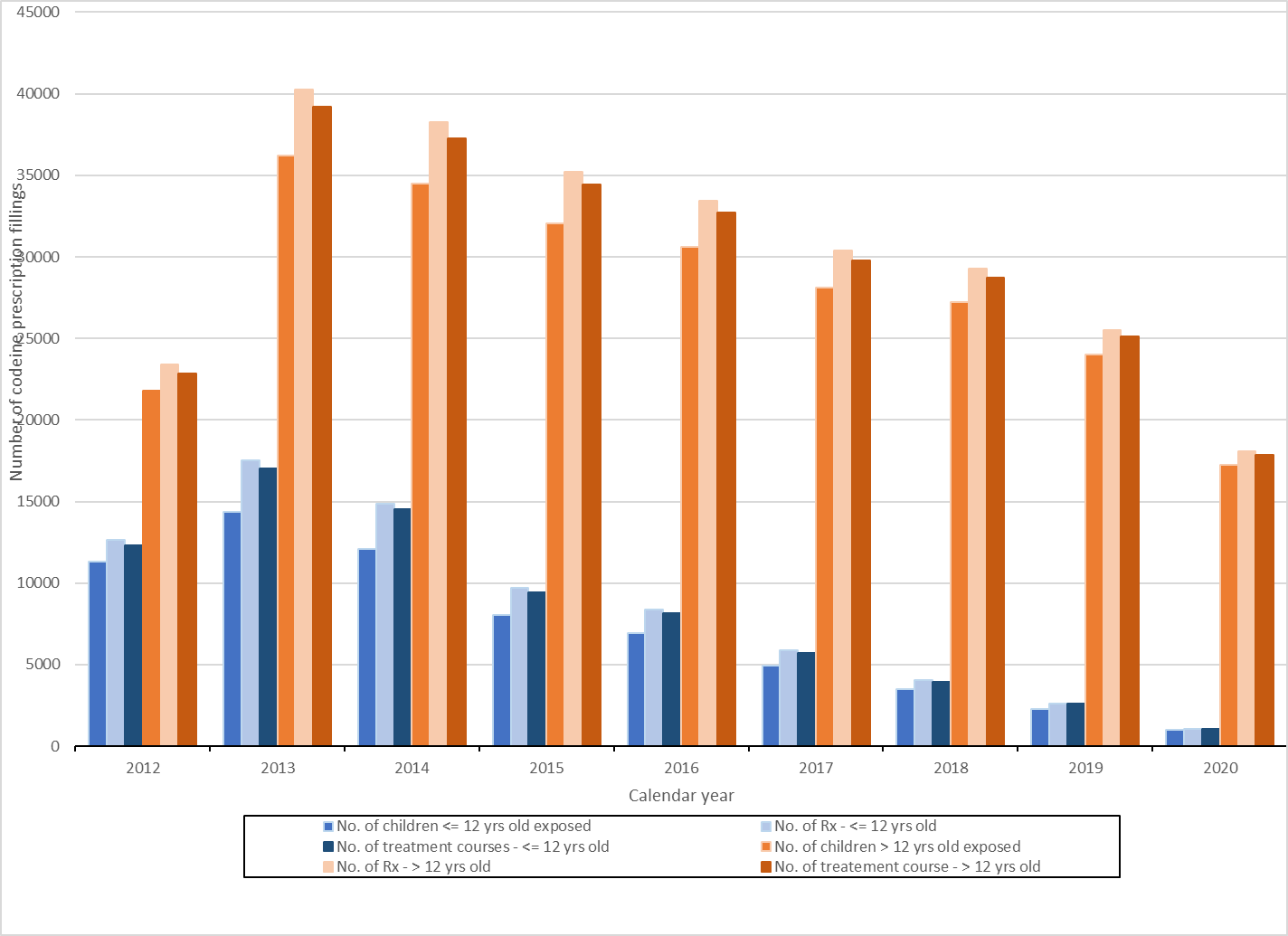
**

1. **Ontario**
2. **Quebec**

**
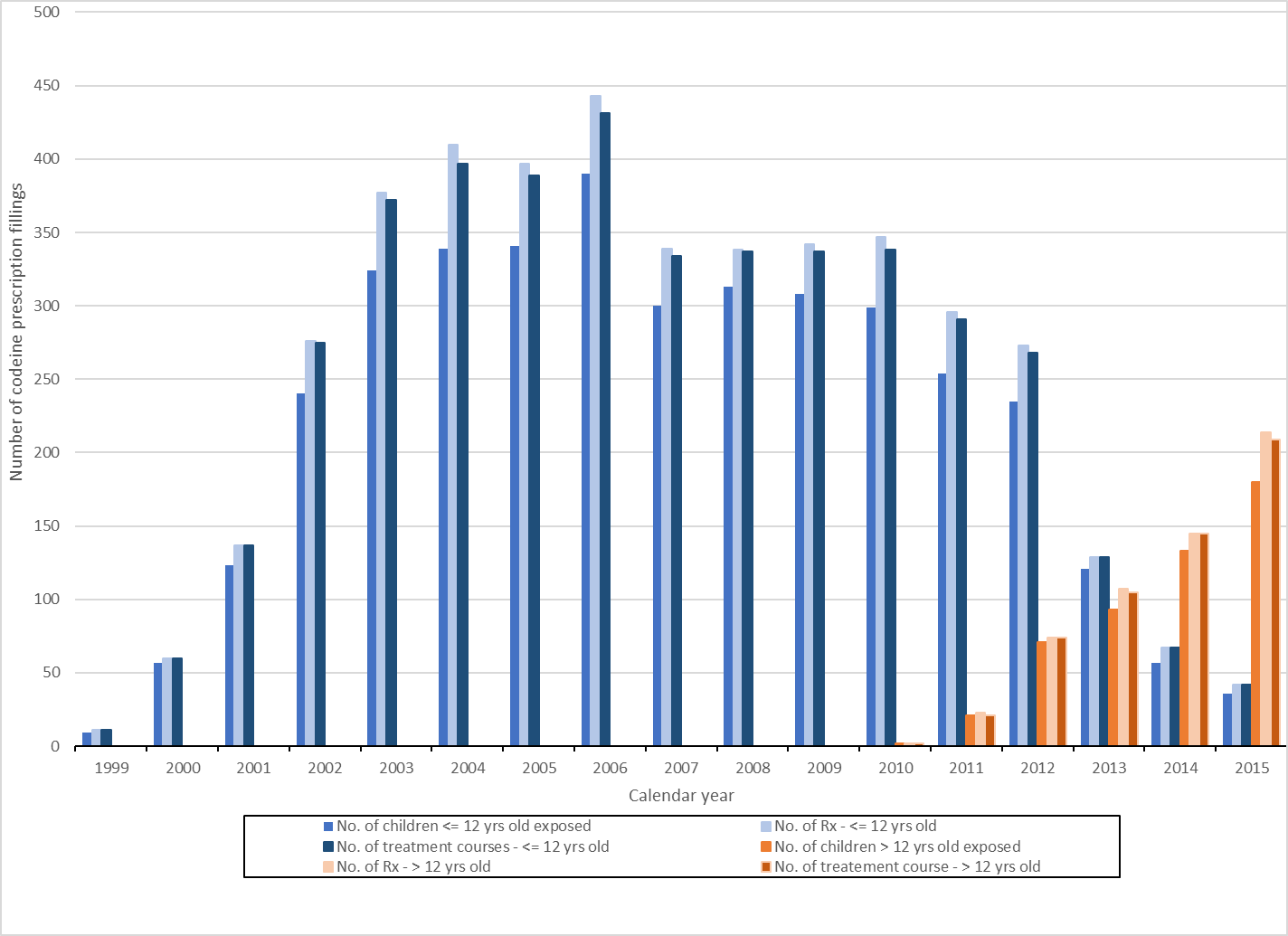
**

**eTable 2.** List of indications for the OMCC fillings with ICD-9 diagnosis codes (3 digits)

| **Indications** | **ICD-9 codes** |
| --- | --- |
| Infectious and parasatic diseases | 001-139 |
| Neoplasms | 140-239 |
| Endocrine, nutritional, and metabolic diseases | 240-279 |
| Diseases of the blood and blood-forming organs | 280-289 |
| Mental disorders | 290-319 |
| Diseases of central nervous system (CNS) | 320-389 |
| Diseases of circulatory system | 390-459 |
| Diseases of respiratory system | 460-519 |
| Diseases of digestive system | 520-579 |
| Diseases of the genitourinary system | 580-629 |
| Diseases of skin and subcutaneous tissue | 680-709 |
| Diseases of the musculoskeletal system | 710-739 |
| Injury and poisoning | 800-999 |
| Other | Other ICD-9 codes |


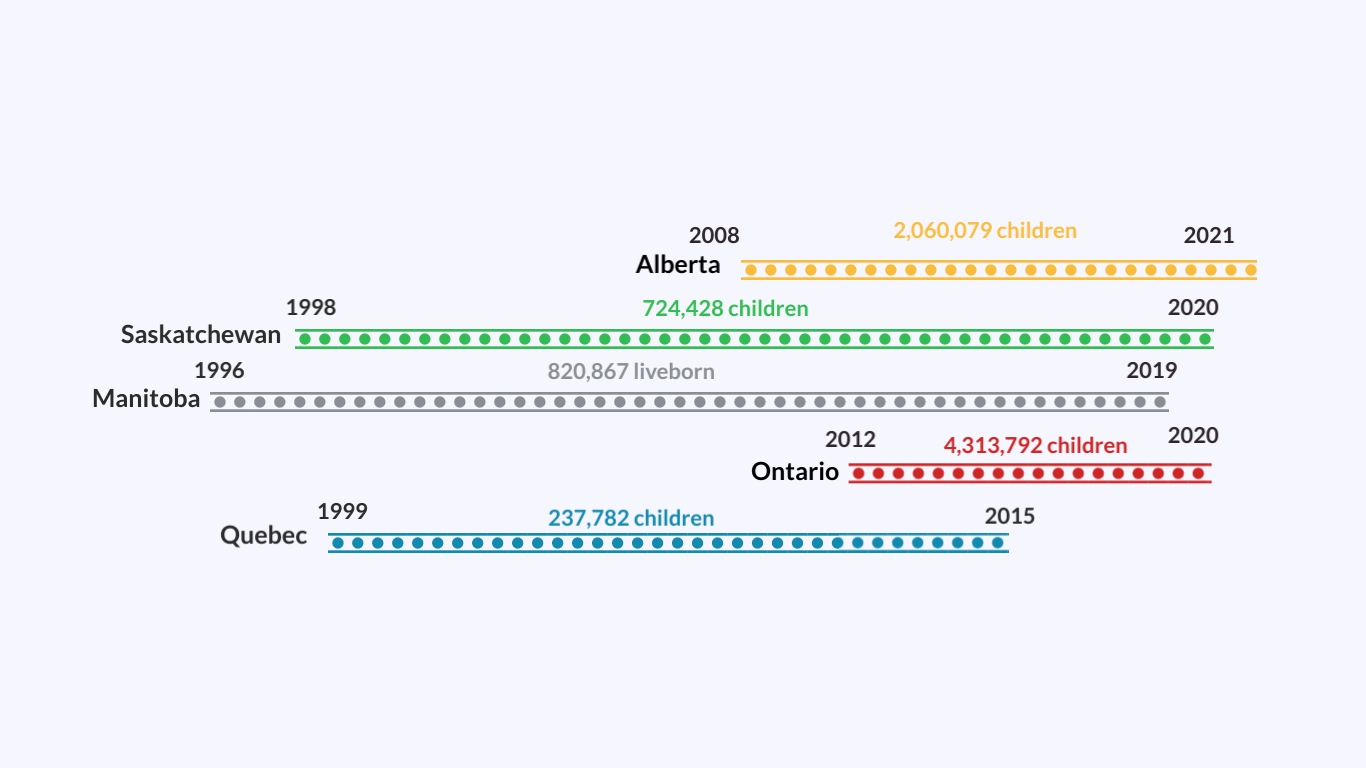
**eFigure 2.** Study periods and total children included in each province.

**eTable 3.** List of DIN numbers of the study medications

| **Active ingredient(s)** | **DIN number** | **Dosage form** | **Strength** |
| --- | --- | --- | --- |
| **Codeine** |  |  |  |
| Codeine phosphate | 00050024 | Syrup | 25mg/5ml |
| Codeine phosphate  pseudoephedrine hydrochloride triprolidine hydrochloride | 00068594 | Syrup | 10mg/5ml  30mg/5ml  2mg/5ml |
| Codeine phosphate  pseudoephedrine hydrochloride triprolidine hydrochloride | 00068608 | Tablet | 20mg  60mg  4mg |
| Codeine phosphate  guaifenesin  pseudoephedrine hydrochloride triprolidine hydrochloride | 00068756 | Syrup | 10mg/5ml  100mg/5ml  30mg/5ml  2mg/5ml |
| Codeine phosphate | 00093114 | Syrup | 5mg/ml |
| Codeine phosphate | 00093122 | Tablet | 15mg |
| Codeine phosphate | 00093130 | Tablet | 30mg |
| Codeine phosphate | 00093149 | Tablet | 60mg |
| Acetylsalicylic acid  Caffeine  codeine phosphate | 00095494 | Tablet | 375mg  15mg  8mg |
| Acetylsalicylic acid  Caffeine  codeine phosphate | 00095508 | Tablet | 375mg  15mg  15mg |
| Acetylsalicylic acid  Caffeine  codeine phosphate | 00095516 | Tablet | 375mg  15mg  30mg |
| Acetylsalicylic acid  caffeine citrate  codeine phosphate | 00108103 | Tablet | 375mg  30mg  15mg |
| Acetylsalicylic acid  caffeine citrate  codeine phosphate | 00108162 | Tablet | 375mg  30mg  8mg |
| Acetylsalicylic acid  caffeine citrate  codeine phosphate  meprobamate | 00108316 | Tablet | 350mg  30mg  15mg  200mg |
| Codeine phosphate  Ipecac  potassium citrate  squill | 00147656 | Syrup | 65mg/30ml  1ml/30ml  975mg/30ml  1.5mg/30ml |
| Acetaminophen  caffeine citrate  codeine phosphate | 00162965 | Tablet | 325mg  30mg  8mg |
| Acetylsalicylic acid  Butalbital  Caffeine  codeine phosphate | 00176192 | Capsule | 330mg  50mg  40mg  15mg |
| Acetylsalicylic acid  butalbital  caffeine  codeine phosphate | 00176206 | Capsule | 330mg  50mg  40mg  30mg |
| Codeine  terpin hydrate | 00179930 | Elixir | 0.23%  1.83% |
| Acetylsalicylic acid  Caffeine  codeine phosphate | 00180041 | Tablet | 375mg  15mg  8mg |
| Acetylsalicylic acid  Caffeine  codeine phosphate | 00219843 | Tablet | 375mg  30mg  30mg |
| Acetaminophen  caffeine citrate  codeine phosphate | 00293490 | Tablet | 325mg  30mg  8mg |
| Acetaminophen/caffeine citrate/codeine phosphate | 00293504 | Tablet | 325mg  30mg  15mg |
| Acetaminophen/caffeine citrate/codeine phosphate | 00293512 | Tablet | 325mg  30mg  30mg |
| Acetaminophen/caffeine citrate/codeine phosphate | 00294942 | Tablet | 325mg/tab  30mg/tab  15mg/tab |
| Acetylsalicylic acid/caffeine citrate/ codeine phosphate | 0194473 | Tablet | 375mg/tab  30mg/tab  30mg/tab |
| Acetaminophen/caffeine citrate/codeine phosphate | 00372323 | Tablet | 300mg  30mg  8mg |
| Acetaminophen/caffeine citrate/codeine phosphate | 00372331 | Tablet | 300mg  30mg  15mg |
| Acetaminophen/caffeine citrate/codeine phosphate | 00372358 | Tablet | 300mg  30mg  30mg |
| Codeine phosphate | 00380571 | Solution | 10mg/5ml |
| Acetaminophen/codeine phosphate | 00396516 | Tablet | 300mg/tab  60mg/tab |
| Acetylsalicylic acid/ codeine phosphate | 00406104 | Tablet | 650mg  30mg |
| Acetylsalicylic acid/ codeine phosphate | 00406112 | Tablet | 325mg  30mg |
| Acetaminophen/caffeine / codeine phosphate | 00425362 | Tablet | 300mg  15mg  8mg |
| Acetaminophen/caffeine / codeine phosphate | 00425370 | Tablet | 300mg/tab  15mg/tab  15mg/tab |
| Acetaminophen/caffeine / codeine phosphate | 00425389 | Tablet | 300mg/tab  15mg/tab  30mg/tab |
| Acetylsalicylic acid/ caffeine /codeine phosphate | 00426865 | Tablet | 400mg  15mg  8mg |
| Acetaminophen/ codeine phosphate | 00440809 | Tablet | 325mg/tab  15mg/tab |
| Ammonium chloride / bromodiphenydramine / codeine phosphate / diphenydramine / potassium guaiacol sulphonate | 00469122 | Liquid | 80mg/5ml  3.75mg/5ml  10mg/5ml  8.75mg/5ml  80mg/5ml |
| Codeine phosphate | 00470651 | Syrup | 5mg/ml |
| Acetaminophen/ codeine phosphate | 00477664 | Tablet | 325mg/tab  30mg/tab |
| Acetaminophen/ codeine phosphate | 00477672 | Tablet | 325mg/tab  60mg/tab |
| Ammonium chloride / codeine phosphate / diphenydramine | 00535230 | Syrup | 125mg/5ml  3.33mg/5ml  12.5mg/5ml |
| Acetylsalicylic acid/ caffeine /codeine phosphate | 00589470 | Tablet | 375mg  30mg  8mg |
| Acetaminophen/ caffeine /codeine phosphate | 00589489 | Tablet | 300mg  30mg  8mg |
| Cocillana / codeine phosphate / euphorbia, senega, squill, wild lettuce | 00593168 | Syrup | 1.36mg/1ml  0.667mg/1ml  3.73mg/1ml  0.566mg/1ml  0.566mg/1ml  1.36mg/1ml |
| Codeine phosphate | 00593435 | Tablet | 15mg |
| Codeine phosphate | 00593451 | Tablet | 30mg |
| Acetaminophen/ caffeine /codeine phosphate | 00599131 | Tablet | 300mg  15mg  8mg |
| Acetylsalicylic acid / butalbital / caffeine /codeine phosphate | 00608181 | Capsule | 330mg  50mg  40mg  30mg |
| Acetylsalicylic acid / butalbital / caffeine /codeine phosphate | 00608203 | Capsule | 330mg  50mg  40mg  15mg |
| Acetaminophen / codeine phosphate | 00608882 | Tablet | 300mg  30mg |
| Acetaminophen / chlorzoxazone / codeine phosphate | 00614920 | Tablet | 300mg  250mg  8mg |
| Acetaminophen/ caffeine /codeine phosphate | 00615528 | Tablet | 300mg  15mg  8mg |
| Acetaminophen/ caffeine /codeine phosphate | 00617059 | Tablet | 300mg  15mg  8mg |
| Acetaminophen/ codeine phosphate | 00621463 | Tablet | 300mg  60mg |
| Acetaminophen/ caffeine /codeine phosphate | 00630594 | Tablet | 500mg  15mg  8mg |
| Acetaminophen/ caffeine /codeine phosphate | 00653233 | Tablet | 300mg  15mg  8mg |
| Acetaminophen/ caffeine /codeine phosphate | 00653241 | Tablet | 300mg  15mg  15mg |
| Acetaminophen/ caffeine /codeine phosphate | 00653276 | Tablet | 300mg  15mg  30mg |
| Acetaminophen/ caffeine /codeine phosphate | 00656119 | Tablet | 300mg  15mg  8mg |
| Acetaminophen/ codeine phosphate | 00666130 | Tablet | 300mg  30mg |
| Acetaminophen/ codeine phosphate | 00666149 | Tablet | 300mg  60mg |
| Acetaminophen/ codeine phosphate | 00685143 | Elixir | 160mg/5ml  8mg/5ml |
| Acetaminophen/ caffeine /codeine phosphate | 00687200 | Tablet | 300mg  15mg  15mg |
| Acetaminophen/ caffeine /codeine phosphate | 00687219 | Tablet | 300mg  15mg  30mg |
| Acetylsalicylic acid/ caffeine /codeine phosphate | 00688851 | Tablet | 325mg  15mg  8mg |
| Ammonium chloride / codeine phosphate / diphenhydramine hydrochloride | 00690074 | Syrup | 125mg/5ml  3.33mg/5ml  12.5mg/5ml |
| Acetylsalicylic acid/ caffeine /codeine phosphate | 00693952 | Tablet | 325mg  15mg  8mg |
| Acetylsalicylic acid/ aluminum hydroxide / caffeine /codeine phosphate / magnesium hydroxide | 00693979 | Tablet | 325mg  35mg  15mg  8mg  70mg |
| Acetaminophen/ caffeine /codeine phosphate | 00706221 | Tablet | 325mg  15mg  8mg |
| Acetaminophen/ caffeine /codeine phosphate | 00706515 | Tablet | 300mg  15mg  15mg |
| Acetaminophen/ caffeine /codeine phosphate | 00706523 | Tablet | 300mg  15mg  30mg |
| Acetaminophen/ caffeine /codeine phosphate | 00708712 | Tablet | 500mg  15mg  8mg |
| Acetaminophen/ caffeine /codeine phosphate / diphenylpyraline / phenylpropanolamine | 00763527 | Tablet | 325mg  32.4mg  8mg  2mg  25mg |
| Codeine phosphate | 00779458 | Tablet | 15mg |
| Codeine phosphate | 00779466 | Tablet | 30mg |
| Codeine phosphate | 00779474 | Syrup | 5mg/ml |
| Codeine phosphate | 00779482 | Liquid | 10mg/ml |
| Ammonium acetate / codeine phosphate / gumweed / menthol / squill | 00779539 | Syrup | 0.66ml/5ml  10mg/5ml  0.02ml/5ml  1.5mg/5ml  1ml/5ml |
| Acetaminophen/ codeine phosphate | 00789828 | Tablet | 300mg  30mg |
| Ammonium chloride / codeine phosphate / diphenydramine hydrochloride | 00792160 | Syrup | 125mg/5ml  3.3mg/5ml  12.5mg/5ml |
| Codeine phosphate / pseudoephedrine hydrochloride / triprolidine | 00809144 | Liquid | 10mg/5ml  30mg/5ml  2mg/5ml |
| Chlorpheniramine / codeine / ephedrine / guaiacol carbonate / phenyltoloxamine | 00815136 | Suspension | 3mg/5ml  10mg/5ml  25mg/5ml  20mg/5ml  5mg/5ml |
| Acetaminophen/ codeine phosphate | 00816027 | Elixir | 160mg/5ml  8mg/5ml |
| Acetaminophen / chlorzoxazone / codeine phosphate | 00834319 | Tablet | 300mg  250mg  8mg |
| Chlorpheniramine maleate / codeine | 00842702 | Syrup (extended-release) | .8mg/ml  2mg/ml |
| Acetaminophen/ caffeine /codeine phosphate | 00852163 | Tablet | 325mg  15mg  8mg |
| Codeine phosphate / guaifenesin / pseudoephedrine hydrochloride | 00889873 | Syrup | 3.3mg/5ml  100mg/5ml  30mg/5ml |
| Codeine phosphate / pheniramine maleate / phenylpropanolamine hydrochloride / pyrilamine maleate | 01906682 | Syrup | 5mg/5ml  6.25mg/5ml  12.5mg/5ml  6.25mg/5ml |
| Acetylsalicylic acid / caffeine /codeine phosphate | 01908596 | Tablet | 325mg  16mg  8mg |
| Acetylsalicylic acid /codeine phosphate / phenobarbital | 01909290 | Capsule | 325mg/cap  16.2mg/cap  16.2mg/cap |
| Acetylsalicylic acid /codeine phosphate / phenobarbital | 01909304 | Capsule | 325mg/cap  64.8mg/cap  16.2mg/cap |
| Acetylsalicylic acid /codeine phosphate / phenobarbital | 01909312 | Capsule | 325mg/cap  32.4mg/cap  16.2mg/cap |
| Acetylsalicylic acid / caffeine /codeine phosphate | 01922246 | Tablet | 325mg  15mg  8mg |
| Codeine phosphate / pheniramine maleate / phenylpropanolamine hydrochloride / pyrilamine maleate | 01925954 | Syrup | 15mg/5ml  12.5mg/5ml  25mg/5ml  12.5mg/5ml |
| Acetylsalicylic acid / caffeine /codeine phosphate | 01933515 | Tablet | 325mg  32mg  8mg |
| Brompheniramine maleate / codeine phosphate / phenylephrine hydrochloride / phenylpropanolamine hydrochloride | 01934694 | Syrup | 2mg/5ml  10mg/5ml  5mg/5ml  5mg/5ml |
| Brompheniramine maleate / codeine phosphate / guaifenesin / phenylephrine hydrochloride / phenylpropanolamine hydrochloride | 01934716 | Syrup | 2mg/5ml  10mg/5ml  100mg/5ml  5mg/5ml  5mg/5ml |
| Codeine phosphate / guaifenesin / pheniramine maleate | 01934732 | Syrup | 3.3mg/5ml  100mg/5ml  7.5mg/5ml |
| Codeine phosphate / guaifenesin / pheniramine maleate | 01934740 | Syrup | 10mg/5ml  100mg/5ml  7.5mg/5ml |
| Acetaminophen / codeine phosphate / methocarbamol | 01934767 | Tablet | 325mg  8mg  400mg |
| Acetylsalicylic acid / codeine phosphate / methocarbamol | 01934775 | Tablet | 325mg  8mg  400mg |
| Acetylsalicylic acid / codeine phosphate / methocarbamol | 01934783 | Tablet | 325mg  16.2mg  400mg |
| Acetylsalicylic acid / codeine phosphate / methocarbamol | 01934791 | Tablet | 325mg  32.4mg  400mg |
| Codeine phosphate / potassium guaiacol sulphonate / promethazine hydrochloride | 01937545 | Liquid | 10mg/5ml  40mg/5ml  5.65mg/5ml |
| Codeine phosphate / phenylephrine hydrochloride / potassium guaiacol sulphonate / promethazine hydrochloride | 01937561 | Liquid | 10mg/5ml  5mg/5ml  44mg/5ml  5mg/5ml |
| Acetaminophen / chlorpheniramine maleate / codeine phosphate / pseudoephedrine hydrochloride | 01938363 | Tablet | 325mg  2mg  8mg  30mg |
| Acetylsalicylic acid / codeine phosphate / methocarbamol | 01941895 | Tablet | 325mg  8mg  400mg |
| Codeine phosphate / guaifenesin / pseudoephedrine hydrochloride | 01944703 | Syrup | 3.3mg/5ml  100mg/5ml  30mg/5ml |
| Alcohol anhydrous / ammonium chloride / codeine phosphate / guaifenesin | 01951564 | Syrup | 3%/5ml  91mg/5ml  10mg/5ml  100mg/5ml |
| Acetylsalicylic acid / codeine phosphate / methocarbamol | 01966367 | Tablet | 325mg  16.2mg  400mg |
| Acetylsalicylic acid / codeine phosphate / methocarbamol | 01966375 | Tablet | 325mg  32.4mg  400mg |
| Acetylsalicylic acid / butalbital / caffeine / codeine phosphate | 01971387 | Capsule | 330mg  50mg  40mg  30mg |
| Acetaminophen / caffeine / codeine phosphate | 01977881 | Tablet | 500mg  15mg  8mg |
| Acetaminophen / caffeine citrate / codeine phosphate | 01996541 | Tablet | 500mg  30mg  8mg |
| Acetaminophen / caffeine citrate / codeine phosphate | 01996568 | Tablet | 500mg  30mg  8mg |
| Acetaminophen / caffeine / codeine phosphate | 01997688 | Tablet | 500mg  15mg  8mg |
| Acetaminophen / codeine phosphate | 01999648 | Tablet | 300mg  30mg |
| Acetaminophen / codeine phosphate | 01999656 | Tablet | 300mg  60mg |
| Codeine phosphate | 02009757 | Tablet | 30mg |
| Codeine phosphate | 02009889 | Tablet | 15mg |
| Codeine phosphate / guaifenesin / pseudoephedrine hydrochloride | 02024810 | Syrup | 3.3mg/5ml  100mg/5ml  30mg/5ml |
| Acetaminophen / caffeine / codeine phosphate | 02025337 | Tablet | 300mg  15mg  8mg |
| Acetaminophen / caffeine / codeine phosphate | 02028174 | Tablet | 300mg  15mg  8mg |
| Acetylsalicylic acid / codeine phosphate / phenobarbital | 02042851 | Capsule | 325mg/cap  16.2mg/cap  16.2mg/cap |
| Acetylsalicylic acid / codeine phosphate / phenobarbital | 02042878 | Capsule | 325mg/cap  32.4mg/cap  16.2mg/cap |
| Acetylsalicylic acid / codeine phosphate / phenobarbital | 02042886 | Capsule | 325mg/cap  64.8mg/cap  16.2mg/cap |
| Acetaminophen / codeine phosphate / doxylamine succinate | 02047667 | Tablet | 325mg  8mg  5mg |
| Codeine phosphate / phenylephrine hydrochloride | 02049465 | Syrup | 15mg/5ml  20mg/5ml |
| Codeine phosphate / guaifenesin / pseudoephedrine hydrochloride / triprolidine hydrochloride | 02053403 | Syrup | 10mg/5ml  100mg/5ml  30mg/5ml  2mg/5ml |
| Codeine phosphate / guaifenesin / pseudoephedrine hydrochloride | 02099748 | Syrup | 3.3mg/5ml  100mg/5ml  30mg/5ml |
| Acetaminophen / caffeine / codeine phosphate | 02143933 | Tablet | 300mg  15mg  8mg |
| Acetaminophen / caffeine / codeine phosphate | 02154234 | Tablet | 300mg  15mg  8mg |
| Codeine phosphate / potassium guaiacol sulphonate / promethazine hydrochloride | 02162180 | Liquid | 10mg/5ml  40mg/5ml  5.65mg/5ml |
| Codeine phosphate | 02163748 | Tablet (extended-release) | 100mg |
| Codeine phosphate | 02163780 | Tablet (extended-release) | 150mg |
| Codeine phosphate | 02163799 | Tablet (extended-release) | 200mg |
| Acetaminophen / codeine phosphate | 02163918 | Tablet | 300mg  60mg |
| Acetaminophen / caffeine / codeine phosphate | 02163926 | Tablet | 300mg  15mg  30mg |
| Acetaminophen / caffeine / codeine phosphate | 02163934 | Tablet | 300mg  15mg  15mg |
| Acetaminophen / codeine phosphate | 02163942 | Elixir | 160mg/5ml  8mg/5ml |
| Codeine phosphate / pseudoephedrine hydrochloride / triprolidine hydrochloride | 02169126 | Syrup | 10mg/5ml  30mg/5ml  2mg/5ml |
| Codeine phosphate / guaifenesin / pseudoephedrine hydrochloride | 02172917 | Syrup | 3.3mg/5ml  100mg/5ml  30mg/5ml |
| Acetaminophen / caffeine / codeine phosphate | 02181061 | Tablet | 300mg  15mg  8mg |
| Acetaminophen / caffeine / codeine phosphate | 02181088 | Tablet | 300mg  15mg  8mg |
| Ammonium chloride / codeine phosphate / diphenhydramine hydrochloride | 02190575 | Syrup | 125mg/5ml  3.3mg/5ml  12.5mg/5ml |
| Codeine phosphate / guaifenesin / pheniramine maleate | 02198630 | Syrup | 10mg/5ml  100mg/5ml  7.5mg/5ml |
| Acetaminophen / codeine phosphate | 02209748 | Tablet | 325mg/tab  30mg/tab |
| Acetylsalicylic acid / butalbital / caffeine / codeine phosphate | 02229734 | Capsule | 330mg  50mg  40mg  30mg |
| Acetylsalicylic acid / butalbital / caffeine / codeine phosphate | 02229735 | Capsule | 330mg  50mg  40mg  15mg |
| Acetaminophen /caffeine / codeine phosphate | 02229962 | Tablet | 325mg  15mg  8mg |
| Codeine phosphate | 02230302 | Tablet (extended-release) | 50mg |
| Acetylsalicylic acid / caffeine / codeine phosphate | 02230426 | Tablet | 375mg  15mg  8mg |
| Acetaminophen / caffeine citrate / codeine phosphate | 02232387 | Tablet | 300mg  30mg  8mg |
| Acetaminophen / caffeine citrate / codeine phosphate | 02232388 | Tablet | 300mg  30mg  15mg |
| Acetaminophen / caffeine citrate / codeine phosphate | 02232389 | Tablet | 300mg  30mg  30mg |
| Acetaminophen / codeine phosphate | 02232658 | Tablet | 300mg  30mg |
| Acetylsalicylic acid / butalbital / caffeine / codeine phosphate | 02232985 | Capsule | 330mg/cap  50mg/cap  40mg/cap  15mg/cap |
| Acetylsalicylic acid / butalbital / caffeine / codeine phosphate | 02232986 | Capsule | 330mg/cap  50mg/cap  40mg/cap  30mg/cap |
| Acetylsalicylic acid / caffeine / codeine phosphate | 02234510 | Tablet | 375mg  30mg  15mg |
| Acetaminophen / caffeine / codeine phosphate | 02236871 | Tablet | 500mg  18mg  8mg |
| Acetaminophen / codeine phosphate / methocarbamol | 02236872 | Tablet | 325mg  8mg  400mg |
| Acetaminophen / caffeine / codeine phosphate | 02237420 | Tablet | 300mg  15mg  8mg |
| Acetaminophen / codeine phosphate / methocarbamol | 02237578 | Tablet | 325mg  8mg  400mg |
| Acetylsalicylic acid / codeine phosphate / methocarbamol | 02237580 | Tablet | 325mg  8mg  400mg |
| Acetaminophen / codeine phosphate / methocarbamol | 02237996 | Tablet | 325mg  8mg  400mg |
| Acetylsalicylic acid / codeine phosphate / methocarbamol | 02237997 | Tablet | 325mg  8mg  400mg |
| Acetylsalicylic acid / caffeine / codeine phosphate | 02238645 | Tablet | 375mg  15mg  30mg |
| Acetylsalicylic acid / caffeine / codeine phosphate | 02238670 | Tablet | 325mg  15mg  8mg |
| Acetylsalicylic acid / caffeine / codeine phosphate | 02238961 | Tablet | 325mg  15mg  8mg |
| Acetaminophen / codeine phosphate / methocarbamol | 02242180 | Tablet | 325mg  8mg  400mg |
| Acetylsalicylic acid / butalbital / caffeine / codeine phosphate | 02242406 | Tablet | 330mg  50mg  40mg  15mg |
| Acetaminophen / codeine phosphate / methocarbamol | 02243047 | Tablet | 325mg  8mg  400mg |
| Codeine phosphate / pseudoephedrine hydrochloride / triprolidine hydrochloride | 02243063 | Syrup | 10mg/5ml  30mg/5ml  2mg/5ml |
| Codeine phosphate | 02243978 | Tablet | 15mg |
| Codeine phosphate | 02243979 | Tablet | 30mg |
| Ammonium chloride / codeine phosphate / diphenhydramine hydrochloride | 02244010 | Liquid | 125mg/5ml  3.33mg/5mg  12.5mg/5ml |
| Codeine phosphate / guaifenesin / pseudoephedrine hydrochloride | 02244011 | Liquid | 3.3mg/5ml  100mg/5mg  30mg/5ml |
| Brompheniramine maleate / codeine phosphate / phenylephrine hydrochloride | 02244078 | Syrup | 0.4mg/ml  2mg/ml  1mg/ml |
| Brompheniramine maleate / codeine phosphate / guaifenesin / phenylephrine hydrochloride | 02244079 | Syrup | 0.4mg/ml  2mg/ml  20mg/ml  1mg/ml |
| Brompheniramine maleate / codeine phosphate / phenylephrine hydrochloride | 02245019 | Syrup | 125mg/5ml  3.3mg/5ml  12.5mg/5ml |
| Ammonium chloride / codeine phosphate / diphenhydramine hydrochloride | 02245592 | Syrup | 125mg/5ml  3.3mg/5ml  12.5mg/5ml |
| Acetylsalicylic acid / caffeine / codeine phosphate | 02245670 | Tablet | 375mg  15mg  8mg |
| Codeine phosphate / guaifenesin / pseudoephedrine hydrochloride | 02245709 | Syrup | 3.3mg/5ml  100mg/5mg  30mg/5ml |
| Acetaminophen/ caffeine / codeine phosphate | 02246085 | Tablet | 300mg  15mg  8mg |
| Acetylsalicylic acid/ caffeine / codeine phosphate | 02246103 | Tablet | 375mg  15mg  8mg |
| Acetaminophen/ caffeine / codeine phosphate | 02248817 | Tablet | 325mg  15mg  8mg |
| Acetaminophen/ codeine phosphate / methocarbamol | 02248977 | Tablet | 325mg  8mg  400mg |
| Acetaminophen/ codeine phosphate / methocarbamol | 02250551 | Tablet | 325mg  8mg  400mg |
| Acetylsalicylic acid/ caffeine / codeine phosphate | 02250683 | Tablet | 375mg  15mg  8mg |
| Acetaminophen/ caffeine / codeine phosphate | 02251914 | Tablet | 300mg  15mg  8mg |
| Acetaminophen/ codeine phosphate | 02254263 | Tablet | 300mg  60mg |
| Acetaminophen/ codeine phosphate | 02254271 | Tablet | 300mg  30mg |
| Acetaminophen/ caffeine / codeine phosphate | 02254700 | Tablet | 300mg  15mg  8mg |
| Codeine phosphate / guaifenesin / pseudoephedrine hydrochloride | 02258099 | Syrup | 3.3mg/5ml  100mg/5mg  30mg/5ml |
| Acetaminophen/ codeine phosphate / methocarbamol | 02258978 | Tablet | 325mg  8mg  400mg |
| Acetaminophen/ caffeine / codeine phosphate | 02259001 | Tablet | 500mg  15mg  8mg |
| Acetaminophen/ caffeine / codeine phosphate | 02265052 | Tablet | 300mg  15mg  8mg |
| Acetaminophen/ caffeine / codeine phosphate | 02271834 | Tablet | 325mg  15mg  8mg |
| Acetaminophen/ caffeine / codeine phosphate | 02277816 | Tablet | 300mg  15mg  8mg |
| Acetaminophen/ codeine phosphate | 02290359 | Tablet | 300mg  30mg |
| Acetaminophen/ codeine phosphate | 02298708 | Elixir | 160mg/5ml  8mg/5ml |
| Acetylsalicylic acid / caffeine / codeine phosphate | 02303299 | Tablet | 375mg  15mg  8mg |
| Codeine phosphate / guaifenesin / pseudoephedrine hydrochloride | 02322196 | Syrup | 3.3mg/5ml  100mg/5mg  30mg/5ml |
| Acetaminophen / caffeine / codeine phosphate | 02336472 | Tablet | 300mg  15mg  8mg |
| Acetaminophen/ caffeine / codeine phosphate | 02387255 | Tablet | 300mg  15mg  8mg |
| Acetaminophen/ caffeine / codeine phosphate | 02439220 | Tablet | 500mg  15mg  8mg |
| Acetaminophen/ caffeine / codeine phosphate | 02453703 | Tablet | 300mg  15mg  8mg |
| Ammonium chloride / codeine phosphate / diphenhydramine hydrochloride | 02486903 | Syrup | 125mg/5ml  3.3mg/5mg  12.5mg/5ml |


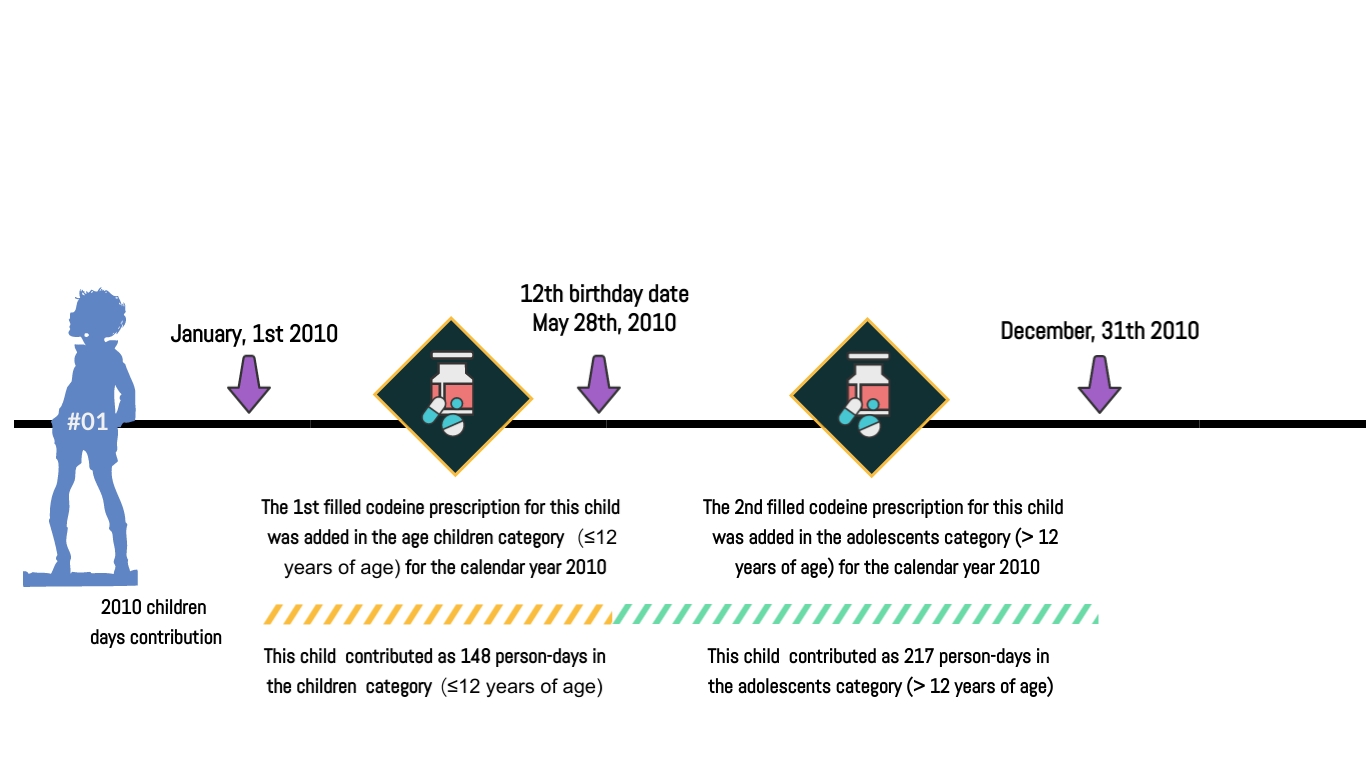
**eFigure 3.** Example of prevalence of codeine exposure and person/days contribution in 2010 for participant #01.


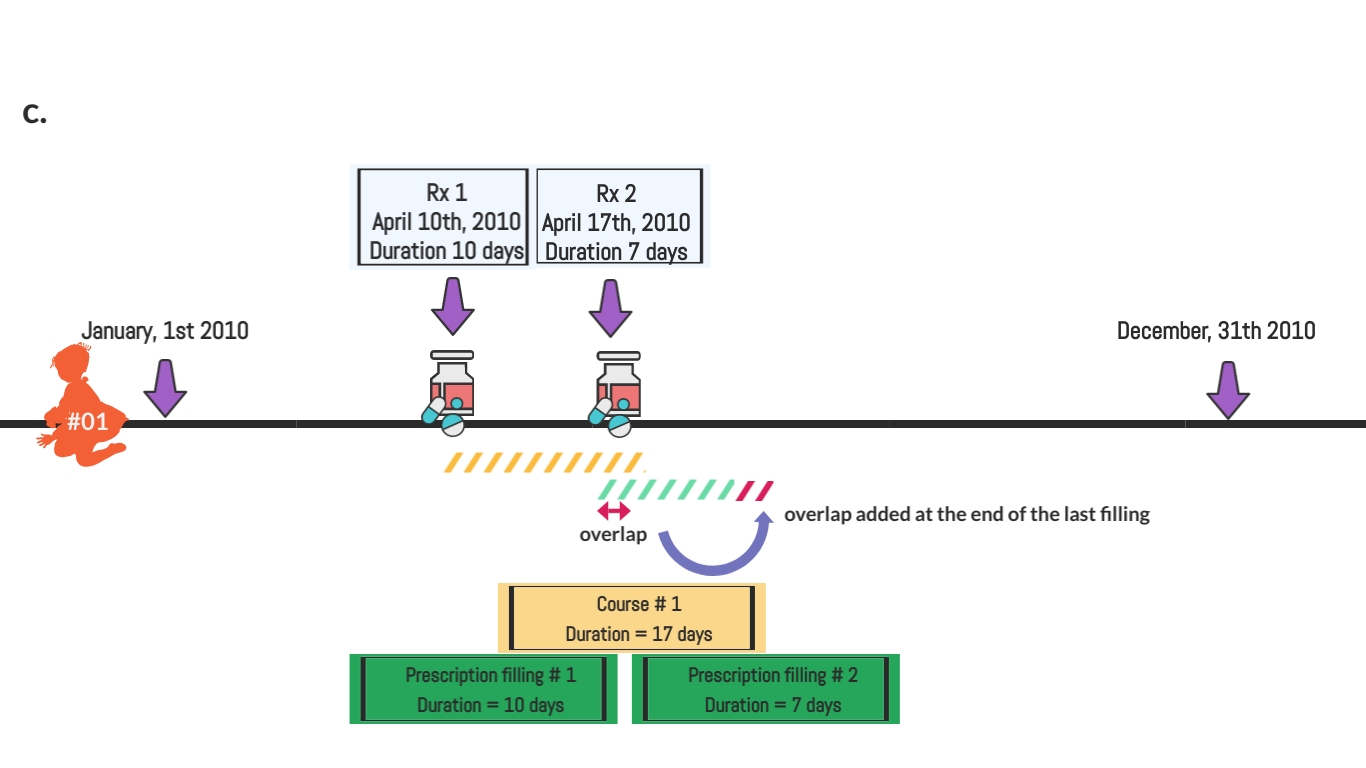

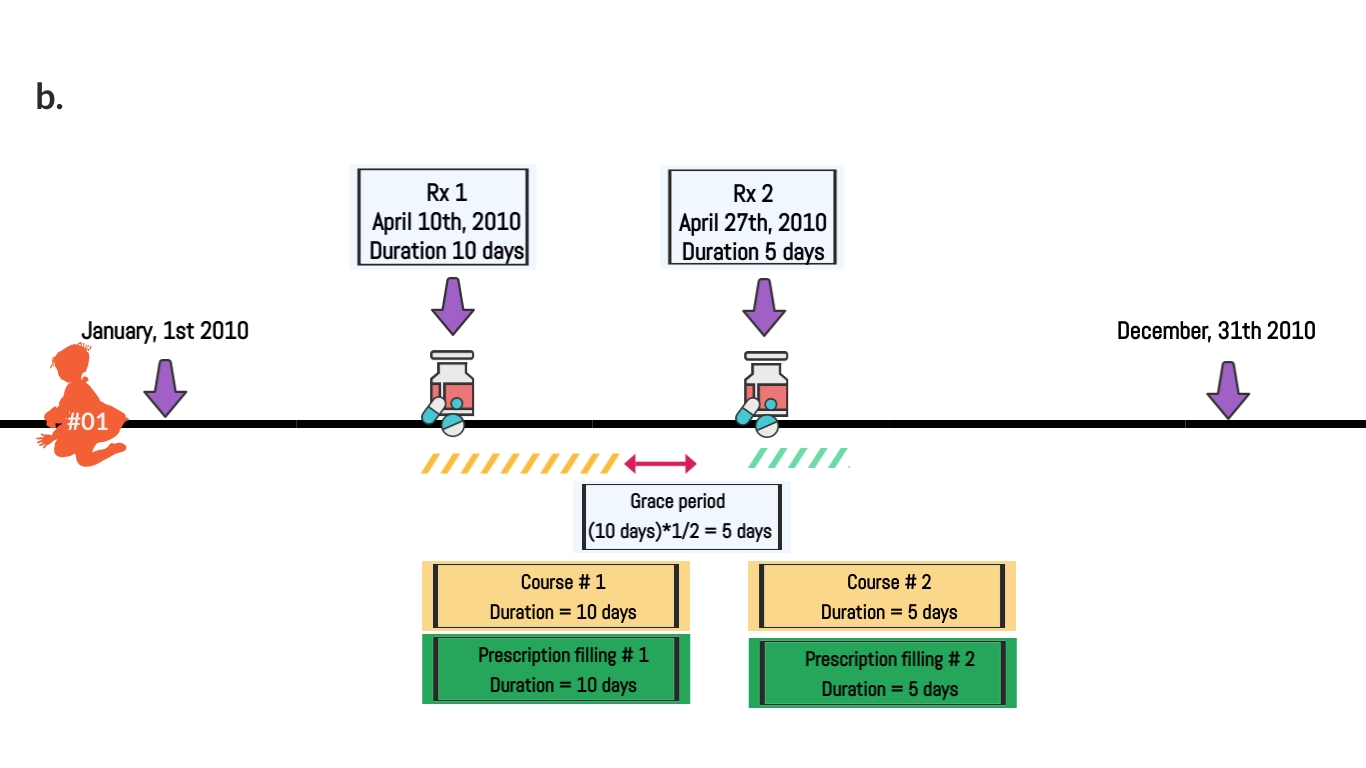

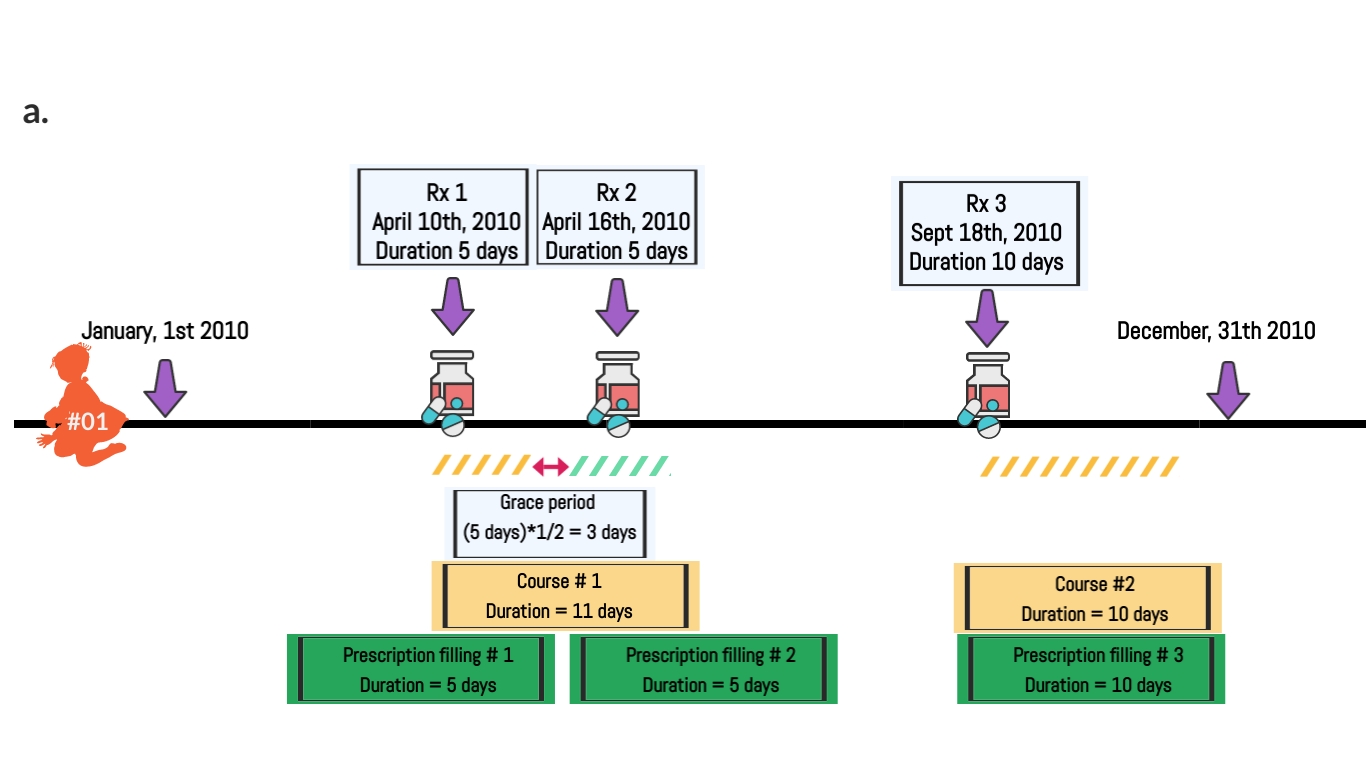
**eFigure 4.** Examples of differences between prescription fillings and treatment course a

1. Information retrieved from [https://www.canada.ca/en/health-canada/services/substance-use/problematic-prescription-drug-u se/opioids/data-surveillance-research/harms-deaths.html](https://www.canada.ca/en/health-canada/services/substance-use/problematic-prescription-drug-u%20se/opioids/data-surveillance-research/harms-deaths.html) on April 4, 2019. [↑](#footnote-ref-1)
2. Gaither JR, Leventhal JM et al. National trends in hospitalizations for opioid poisonings among children and adolescents, 1997 to 2012. JAMA Pediatr. 2016;170(12):1195-1201) [↑](#footnote-ref-2)
3. Information retrieved from [↑](#footnote-ref-3)
4. [↑](#footnote-ref-4)
